# Half logistic exponentiated inverse Rayleigh distribution: Properties and application to life time data

**DOI:** 10.1371/journal.pone.0310681

**Published:** 2025-01-16

**Authors:** Juma Salehe Kamnge, Manoj Chacko

**Affiliations:** 1 Department of Mathematics and Statistics, Mzumbe University, Morogoro, Tanzania; 2 Department of Statistics, University of Kerala, Trivandrum, India; UDOM: The University of Dodoma, TANZANIA, UNITED REPUBLIC OF

## Abstract

This paper presents a novel extension of the exponentiated inverse Rayleigh distribution called the half-logistic exponentiated inverse Rayleigh distribution. This extension improves the flexibility of the distribution for modeling lifetime data for both monotonic and non-monotonic hazard rates. The statistical properties of the half-logistic exponentiated inverse Rayleigh distribution, such as the quantiles, moments, reliability, and hazard function, are examined. In particular, we provide several techniques to estimate the half-logistic exponentiated inverse Rayleigh distribution parameters: weighted least squares, Cramér-Von Mises, maximum likelihood, maximum product spacings and ordinary least squares methods. Moreover, numerical simulations were performed to evaluate these estimation methods for both small and large samples through Monte Carlo simulations, and the finding reveals that the maximum likelihood estimation was the best among all estimation methods since it comprises small mean square error compared to other estimation methods. We employ real-world lifetime data to demonstrate the performance of the newly generated distribution compared to other distributions through practical application. The results show that the half-logistic exponentiated inverse Rayleigh distribution performs better than alternative versions of the Rayleigh distributions.

## 1 Introduction

The practice of introducing additional parameters to create new distributions has become increasingly prevalent in statistical theory in recent decades [1]. By considering the relevance of probability theory and statistics, there is often a need for a generalised lifetime model that can effectively analyse data in the field of survival and reliability analysis [2]. Anwar and Bibi [3] derived a novel probability distribution by employing the generalized weibull distribution function on the half logistic family of distribution. Alzaatreh et al. [4] introduced T-X family of continuous distributions by interchanging the probability density function (pdf) of any continuous random variable with the pdf of beta distribution. Lee et al. [5] devised a method for producing continuous distributions with a single variable. Jones [6] employed the Beta distribution to analyse the set of distributions introduced by Eugene et al. [7].

In line with that, this study employed the half logistic transformation method to the exponentiated inverse Rayleigh (EIR) distribution as the base model since this distribution was derived from the Rayleigh distribution, which was used in the field of engineering, physics, and medical side in modeling the survival and reliability data. Additionally, this base model was proven to be flexible in the sense that when the shape of EIR is one, this distribution returns to inverse Rayleigh (IR), which was the base for EIR [8]. Furthermore, this base distribution for this current study, i.e., EIR, was found to be useful in the area of quality control to assess the quality of coating weights of iron sheets data [8]. Despite its usefulness, the EIR has the following limitation: it is not able to accurately model complex data structures especially modeling moderately right-skewed or near-symmetrical lifetime data, more specifically this distribution is able to model only non-monotonically hazard rate function. For instance, the Exponential and Weibull distributions are unable to accurately represent real data that follows a non-monotonic failure rate function [9]. Owning that, this paper aims to contribute to the generation of a new probability distribution that models the data with both monotonically and non-monotonically hazard rate functions since data usually come from the field of survival and reliability analysis, especially from engineering, geology, education, economics, and health have monotonic and non-monotonic behavior.

In the Recent past, Cordeiro et al. [10] suggested a new technique, called half-logistic transformation, by including an additional parameter in the life time model. The primary purpose of this family was to utilize the non-symmetrical behavior of the parent distribution. Let *X* is a continuous random variable with cumulative distribution function (cdf) *F*(*x*) then the cdf of type I half-logistic family of distributions is given by

$$\begin{array}{c} F\left(x\right)=\frac{1-{\left(1-G\left(x\right)\right)}^{\lambda }}{1+{\left(1-G\left(x\right)\right)}^{\lambda }},\;\;\;\lambda >0. \end{array}$$ (1)

While the pdf is given by

$$\begin{array}{c} f\left(x\right)=\frac{2\lambda g\left(x\right){\left(1-G\left(x\right)\right)}^{\lambda -1}}{{\left(1+{\left(1-G\left(x\right)\right)}^{\lambda }\right)}^{2}},\;\;\;\lambda >0, \end{array}$$ (2)

where *g*(*x*) and *G*(*x*) are the pdf and cdf of base distribution respectively.

The half-logistic transformation has been used by many researchers; for example, Anwar and Bibi [3] explored the new probability distributions by applying the generalized Weibull distribution to the half-logistic family of distributions. Moreover, using the same half-logistic transformation, Moakofi et al. [11] produced a half-logistic log-logistic Weibull distribution, and Dhungana et al. [12] produced half-logistic inverted Weibull distribution.

The main aim of this paper is to produce a new probability distribution by using the half logistic family of distributions. In this paper, we consider the exponentiated inverse Rayleigh distribution by Rao and Mbwambo [8] as the baseline distribution. A random variable *X* is said to follow an exponetiated inverse Rayleigh distribution if it possesses the following pdf and cdf respectively.

$$\begin{array}{c} g\left(x\right)=\frac{2\alpha \sigma^{2}e^{-\frac{\sigma^{2}}{x^{2}}}{\left(1-e^{-\frac{\sigma^{2}}{x^{2}}}\right)}^{\alpha -1}}{x^{3}},\;x,\;\sigma ,\;\alpha >0, \end{array}$$ (3)

and

$$\begin{array}{c} G\left(x\right)=1-{\left(1-e^{-{\left(\frac{\sigma }{x}\right)}^{2}}\right)}^{\alpha }. \end{array}$$ (4)

The distribution thus obtained is called half logistic exponentiated inverse Rayleigh distribution (HLEIRD). The new distribution is able to model data with both monotonically and non-monotonically hazard rate function. In addition, this article attempt to estimate the parameters of the model by applying different estimations methods, so this study aims to develop a guideline for choosing the best estimation method for the HLEIRD which would be of profound interest to applied statisticians. The choice of the methods of estimation varies among the users and area of applications. The present study is unique because so far no attempt has been made to develop half logistic exponentiated inverse Rayleigh distribution and to compare the aforementioned methods of estimation for parameters. The rest of the paper is organised as follows: In section 2, pdf, cdf, survival function, and hazard function of HLEIRD are introduced together with their graphs. In section 3>, statistical and mathematical properties such as quantile function, median, mode, moment generating function, moments, and order statistics are obtained. Furthermore, in section 4, different estimation methods such as maximum likelihood estimation, Maximum product spacing estimation, least squares estimation, cramér-von mises estimation, and weighted least squares estimation are considered. In section 5, simulation studies are performed to assess the efficiency of different methods for estimation. In section 6, a real data is applied to prove the flexibility and suitability of the model, and lastly, in section 7, which summarises the conclusion of the study.

## 2 Half logistic exponentiated inverse Rayleigh distribution

By applying the cdf of the exponentiated inverse Rayleigh distribution defined in (4) to the cdf defined in (1), we obtain the cdf for the HLEIRD and is given by

$$\begin{array}{c} F\left(x\right)=\frac{1-{\left(1-e^{-{\left(\frac{\sigma }{x}\right)}^{2}}\right)}^{\alpha \lambda }}{1+{\left(1-e^{-{\left(\frac{\sigma }{x}\right)}^{2}}\right)}^{\alpha \lambda }}\;,x>0;\;\alpha >0,\lambda >0,\sigma >0. \end{array}$$ (5)

The pdf corresponding to the above cdf is given by

$$\begin{array}{c} f\left(x\right)=\frac{(4\lambda \alpha \sigma^{2})e^{-{\left(\frac{\sigma }{x}\right)}^{2}}{\left(1-e^{-{\left(\frac{\sigma }{x}\right)}^{2}}\right)}^{\alpha \lambda -1}}{x^{3}{\left(1+{\left(1-e^{-{\left(\frac{\sigma }{x}\right)}^{2}}\right)}^{\alpha \lambda }\right)}^{2}}\;,\alpha >0,\lambda >0,\sigma >0,x>0. \end{array}$$ (6)

**Fig 1:**
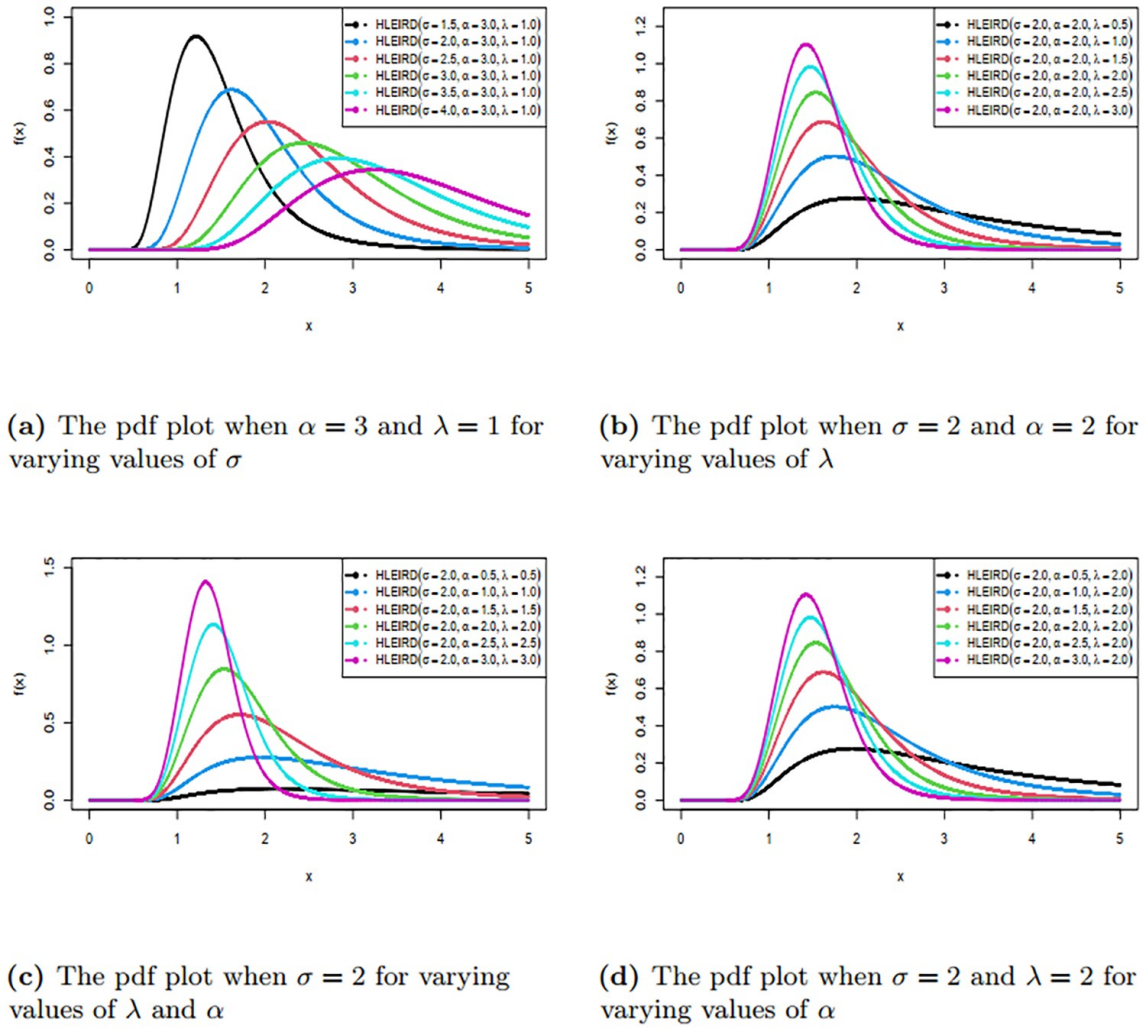
Probability density function of HLEIRD with different parameters.

**Fig 2:**
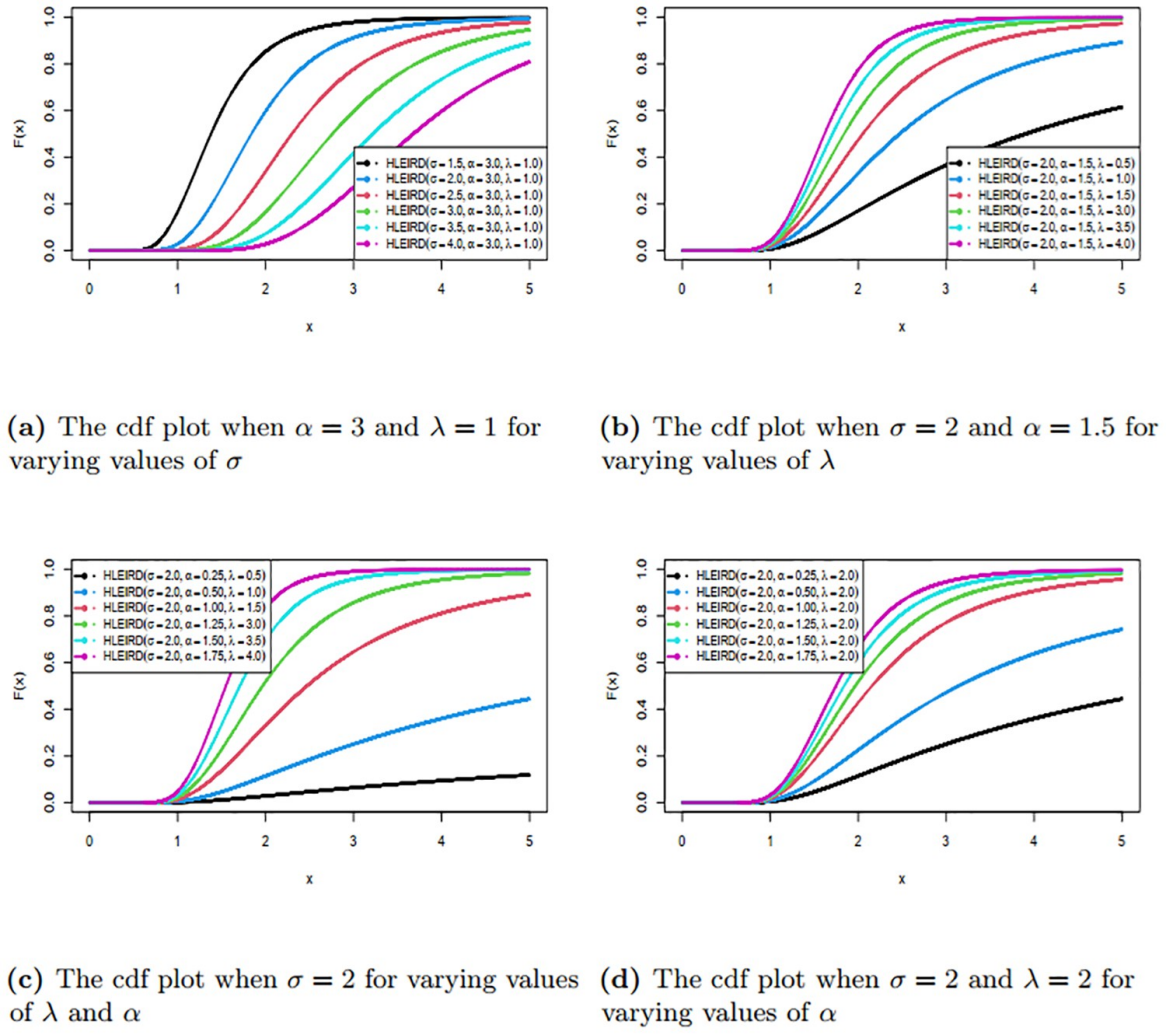
Cumulative density function of HLEIRD with different parameters.

We have drawn the graphs of pdf and cdf of HLEIRD for the different parameter values and are given in Figs 1 and 2. Figs 1 and 2 demonstrate that the HLEIRD exhibits greater flexibility when dealing with various shapes, including symmetrical shapes as well as left and right-skewed shapes. This characteristic makes it suitable over wide range of lifetime data.

The survival function and hazard rate function, for HLEIRD are respectively given by:

$$\begin{array}{c} S\left(x\right)=\frac{2{\left(1-e^{-{\left(\frac{\sigma }{x}\right)}^{2}}\right)}^{\alpha \lambda }}{1+{\left(1-e^{-{\left(\frac{\sigma }{x}\right)}^{2}}\right)}^{\alpha \lambda }} \end{array}$$ (7)

and

$$\begin{array}{c} h\left(x\right)=\frac{(2\lambda \alpha \sigma^{2})e^{-{\left(\frac{\sigma }{x}\right)}^{2}}{\left(1-e^{-{\left(\frac{\sigma }{x}\right)}^{2}}\right)}^{-1}}{x^{3}(1+{\left(1-e^{-{\left(\frac{\sigma }{x}\right)}^{2}}\right)}^{\alpha \lambda })}. \end{array}$$ (8)

**Fig 3:**
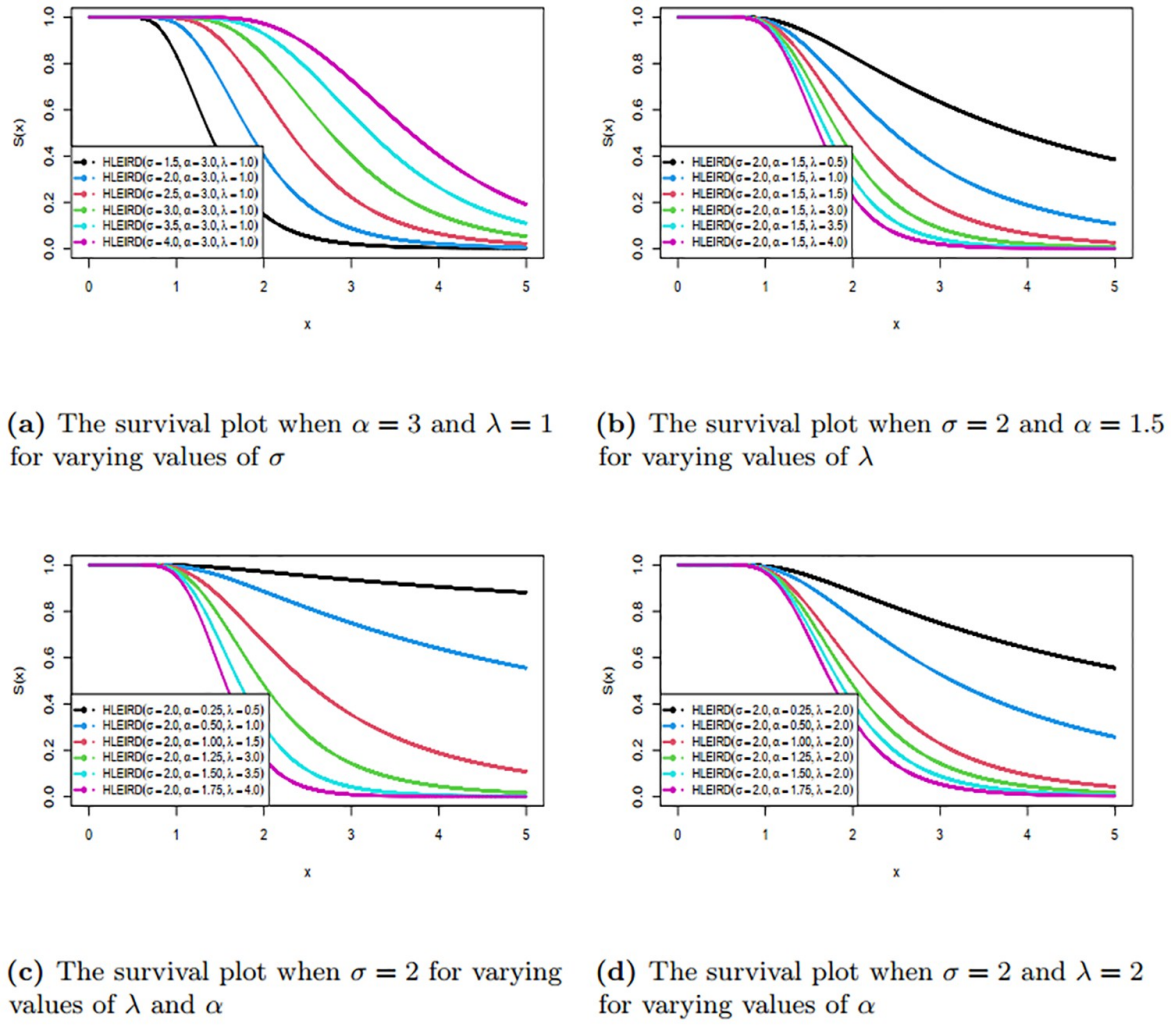
Survival function of HLEIRD with different parameter values.

**Fig 4:**
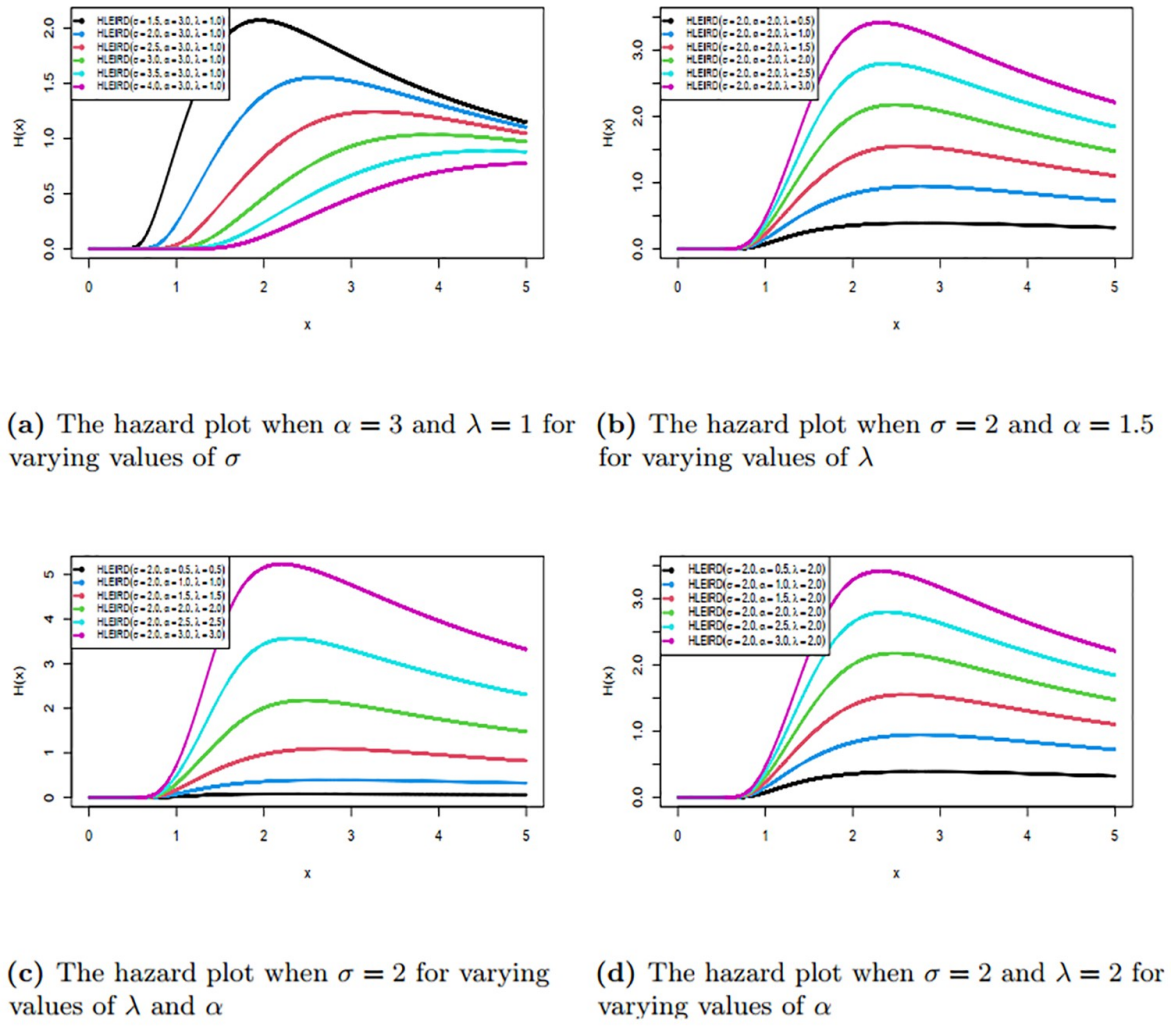
Curve of hazard function of HLEIRD with different values of parameter.

We have also drawn the graphs of survival function and hazard rates function of HLEIRD for different parametric values and are given in Figs 3 and 4. The Figs 3 and 4 reveal that this family is capable of producing various shapes, such as reverse J curve, reserve S curve, increasing and decreasing curve. For more details see Table 1 below.

**Table 1:** Show the shape of survival and hazard curve for some values of (*σ*, *α*,λ) of HLEIRD.

S/N	Parameters	Explanation
1	*α* ≤0.5	The survival curve depicts decreasing rate shape
2	*σ* = 3, *α* = 3 and λ=1	The survival curve depicts that reverse S curve
3	*σ* = 2, *α* = 3 and λ=2	The Hazard curve depicts increasing rate shape
4	For different values of *σ*, *α* and λ	Hazard curve depicts that reverse J curve

This demonstrates that the HLEIRD family has the potential to effectively accommodate data sets with a wide range of shapes. This implies that distribution is very powerful in modelling survival and reliability data.

## 3 Statistical and mathematical properties

In this section, we furnish some significant statistical and mathematical properties of the HLEIRD such as moments, moment generating function, ordered statistics, mode, quantiles, median, skewness, and kurtosis.

### 3.1 Some stochastic ordering results

The HLEIRD enjoys tractable stochastic ordering results involving the corresponding cdf. From a statistical point of view, such results allow a better comprehension of the roles of the parameters in the fitting ability of the HLEIRD model. The most notable of them are presented below.

**Proposition 1**. If *F*(*x*, *φ*) is the cdf of HLEIRD defined in (5), where *φ* = (λ, *σ*, *α*) then the following inequalities holds:

1) For any *α*_1_ ≥ *α*_2_ and λ, *σ*, *x* > 0, we have *F*(*x*, λ, *α*_1_, *σ*)≥*F*(*x*, λ, *α*_2_, *σ*)
2) For any λ_1_ ≥ λ_2_ and λ, *σ*, *x* > 0, we have *F*(*x*, λ_1_, *α*, *σ*)≥*F*(*x*, λ_2_, *α*, *σ*)
3) For any *σ*_1_ ≥ *σ*_2_ and λ, *α*, *x* > 0, we have *F*(*x*, λ, *α*, *σ*_1_)≥*F*(*x*, λ, *α*, *σ*_2_)

Proof. The proof is based on monotonic arguments with respect to the parameters. We have

$$\frac{\partial F(x;\varphi )}{\partial \alpha }=-\frac{2\lambda \text{log}(1-e^{-\frac{\sigma^{2}}{x^{2}}}){\left(1-e^{-\frac{\sigma^{2}}{x^{2}}}\right)}^{\alpha \lambda }}{{\left({\left(1-e^{-\frac{\sigma^{2}}{x^{2}}}\right)}^{\alpha \lambda }+1\right)}^{2}}<0.$$

This implies that *F*(*x*;*φ*) is strictly decreasing with respect to *α*. Therefore for any *α*_1_ ≥ *α*_2_ and λ, *σ*, *x* > 0, we have *F*(*x*, λ, *α*_1_, *σ*)≥*F*(*x*, λ, *α*_2_, *σ*). With the same methodology, we have

$$\frac{\partial F(x;\varphi )}{\partial \lambda }=-\frac{2\alpha \;\text{log}(1-e^{-\frac{\sigma^{2}}{x^{2}}}){\left(1-e^{-\frac{\sigma^{2}}{x^{2}}}\right)}^{\alpha \lambda }}{{\left({\left(1-e^{-\frac{\sigma^{2}}{x^{2}}}\right)}^{\alpha \lambda }+1\right)}^{2}}<0.$$

This implies that *F*(*x*;*φ*) is strictly decreasing with respect to λ. Therefore for any λ_1_ ≥ λ_2_ and λ, *σ*, *x* > 0, we have *F*(*x*, λ_1_, *α*, *σ*)≥*F*(*x*, λ_2_, *α*, *σ*). Similarly, we have

$$\frac{\partial F(x;\varphi )}{\partial \sigma }=-\frac{4\alpha \lambda \sigma {\left(1-e^{-\frac{\sigma^{2}}{x^{2}}}\right)}^{\alpha \lambda }}{x^{2}\left(e^{\frac{\sigma^{2}}{x^{2}}}-1\right){\left({\left(1-e^{-\frac{\sigma^{2}}{x^{2}}}\right)}^{\alpha \lambda }+1\right)}^{2}}<0.$$

This implies that *F*(*x*;*φ*) is strictly decreasing with respect to *σ*. Therefore for any *σ*_1_ ≥ *σ*_2_ and λ, *α*, *x* > 0, we have *F*(*x*, λ, *α*, *σ*_1_)≥*F*(*x*, λ, *α*, *σ*_2_).

### 3.2 Linear representation

The following result introduces a useful linear representation for the exponentiated pdf of the HLEIRD with power parameter *v* > 0.

**Proposition 2**. Let *v* > 0. Then, *f*(*x*;*φ*)^*v*^ can be expressed as the following series expansion:

$$f{\left(x,\varphi \right)}^{\nu }=\sum_{j,k=0}^{\infty }c_{j,k}\left(\varphi ,\nu \right)g_{k}\left(x,\varphi ,\nu \right)\;,$$

where

cj,k(φ,ν)=4νλνανσ2ν(-1)k(-2νj)(λαj+(λα-1)νk)

and

$$g_{k}\left(x,\varphi ,\nu \right)=x^{-3\nu }e^{-\left(k+\nu \right){\left(\frac{\sigma }{x}\right)}^{2}}\;.$$

**Proof**. From (6) the pdf of HLEIRD is given by

$$\begin{array}{c} f\left(x,\varphi \right)=\frac{(4\lambda \alpha \sigma^{2})e^{-{\left(\frac{\sigma }{x}\right)}^{2}}{\left(1-e^{-{\left(\frac{\sigma }{x}\right)}^{2}}\right)}^{\alpha \lambda -1}}{x^{3}{\left(1+{\left(1-e^{-{\left(\frac{\sigma }{x}\right)}^{2}}\right)}^{\alpha \lambda }\right)}^{2}}\;,\alpha >0,\lambda >0,\sigma >0,x>0. \end{array}$$

where *φ* = (λ, *σ*, *α*). Then

$$\begin{array}{c} f{\left(x,\varphi \right)}^{\nu }=4^{\nu }\alpha^{\nu }\lambda^{\nu }\sigma^{2\nu }x^{-3\nu }e^{-\nu {\left(\frac{\sigma }{x}\right)}^{2}}{\left(1-e^{-{\left(\frac{\sigma }{x}\right)}^{2}}\right)}^{\nu (\lambda \alpha -1)}{\left(1+{\left(1-e^{-{\left(\frac{\sigma }{x}\right)}^{2}}\right)}^{\lambda \alpha }\right)}^{-2\nu }. \end{array}$$

Then by using the generalized binomial series formula given by

(1+Z)-k=∑i=0∞(-ki)Zi.

where |*Z*| < 1, *k* > 0 and (-ki) represents the generalized binomial coefficient defined by

(-ki)=(-k)(-k-1)(-k-2)⋯(-k-i+1)i!,=(-1)ik(k+1)(k+2)⋯(k+i-1)i!,

we get

f(x,φ)ν=4νανλνσ2νx-3νe-ν(σx)2∑j=0∞(-2νj)(1-e-(σx)2)λαj+ν(λα-1),=4νλνανσ2νx-3ν∑j,k=0∞(-1)k(-2νj)(λαj+(λα-1)νk)e-(k+ν)(σx)2. (9)

After rearrangement, we get the desired result. Hence the proof.

By taking *ν* = 1 in (9), we get a useful series expansion for the pdf of the HLEIRD, in the sense that we express a sophisticated function as sums of tractable functions, i.e., *g*_*k*_(*x*, *φ*, *ν*). In particular, we will use it in the coming sections to provide measures and functions which are easy to handle from the analytical and numerical point of views.

### 3.3 Moments of HLEIRD

The *r*^*th*^ raw moment of HLEIRD is given by

$\mu_{r}^{\prime }=E\left(X^{r}\right)=\int_{0}^{\infty }x^{r}f\left(x,\phi \right)\;dx$ . By using the Proposition 2 with *ν* = 1, we obtain the following:

μr′=4λασ2∑j,k=0∞(αλj+αλ-1k)(-1)k∫0∞xr-3e(k+1)(-(σx)2)dx=4λασ2∑j,k=0∞(αλj+αλ-1k)(-1)k[(σ2(k+1))r2Γ(1-r2)2σ2(k+1)]=2λα∑j,k=0∞(αλj+αλ-1k)(-1)k[(σ2(k+1))r2Γ(1-r2)(k+1)]. (10)

The mean of the HLEIRD can be obtained by putting *r* = 1 in (10) and is given by

E(X)=2λα∑j,k=0∞(αλj+αλ-1k)(-1)k[(σ2(k+1))12Γ(12)(k+1)].

From (10) it can be shown that HLEIRD has first moment (mean) only.

### 3.4 Moment generating function

The moment generating function of HLEIRD is given by $M_{X}\left(t\right)=\int_{-\infty }^{\infty }e^{tx}f\left(x,\phi \right)\;dx$. By using the Proposition 2 with *ν* = 1, we have the following

MX(t)=4λασ2∑j,k=0∞(αλj+αλ-1k)(-1)k∫0∞x-3etxe(k+1)(-(σx)2)dx.

By using the expansion

$$e^{tx}=\sum_{r=0}^{\infty }\frac{{\left(tx\right)}^{r}}{r!},$$

we get the following

MX(t)=4λασ2∑j,k,r=0∞(αλj+αλ-1k)(-1)k(t)rr!∫0∞xr-3e-(σx)2(k+1)dx=4λασ2∑j,k,r=0∞(αλj+αλ-1k)(-1)k(t)rr![(σ2(k+1))r2Γ(1-r2)2σ2(k+1)].

Thus the mgf of HLEIRD is given by

MX(t)=2λα∑j,k,r=0∞(αλj+αλ-1k)(-1)k(t)rr![(σ2(k+1))r2Γ(1-r2)(k+1)].

### 3.5 Entropy measures

The entropy of the HLEIRD can be measured in different ways. Here, we focus our attention on the Rényi entropy by [13] and the q-entropy by [14]. For discussions and applications of these two entropy measures, refer [15], and the references therein. The Rényi entropy of the HLEIRD given by, for *δ* ≠ 1

$$I_{\delta }\left(\varphi \right)=\frac{1}{1-\delta }\text{log}[\int_{0}^{\infty }f{\left(x;\varphi \right)}^{\delta }dx].$$

We have

$$\begin{array}{c} \begin{array}{cc} \int_{0}^{\infty }f{\left(x;\varphi \right)}^{\delta }dx & =\sum_{j,k=0}^{\infty }c_{j,k}\left(\varphi ,\delta \right)\int_{0}^{\infty }g_{k}\left(x,\varphi ,\delta \right)\\ & =\sum_{j,k=0}^{\infty }\frac{e_{j,k}\left(\varphi ,\delta \right)}{{\left(1+k\right)}^{\frac{3\delta }{2}-\frac{1}{2}}}dx, \end{array} \end{array}$$ (11)

where

ej,k(φ,δ)=12b1-3δΓ(3δ2-12)cj,k(φ,δ)=12b1-3δΓ(3δ2-12)(-1)k(-2δj)(λαj+(λα-1)δk).

Therefore, by referring (11), one can express I_*δ*_ as:

$$I_{\delta }\left(\varphi \right)=\frac{1}{1-\delta }\text{log}[\sum_{j,k=0}^{\infty }\frac{e_{j,k}\left(\varphi ,\delta \right)}{{\left(1+k\right)}^{\frac{3\delta }{2}-\frac{1}{2}}}].$$

Then, the q-entropy of the HLEIRD is defined by, for *q* ≠ 1

$$H_{q}\left(\varphi \right)=\frac{1}{1-q}\text{log}[1-\int_{0}^{\infty }f{\left(x;\varphi \right)}^{q}dx].$$

Therefore, by using (11), with a similar approach, we get

$$H_{q}\left(\varphi \right)=\frac{1}{1-q}[1-\sum_{j,k=0}^{\infty }\frac{e_{j,k}\left(\varphi ,q\right)}{{\left(1+k\right)}^{\frac{3q}{2}-\frac{1}{2}}}].$$

### 3.6 Order statistics

Moments of order statistics have great role in quality control testing and reliability to predict time to fail of a certain item by considering few early failures. Suppose *X*_1:*m*_ < *X*_2:*m*_, ⋯, <*X*_*m*: *m*_ are ordered statistics of a random sample size *m* drawn from HLEIRD with cdf *F*_*X*_(*x*) and pdf *f*_*X*_(*x*) then the pdf of *X*_*k*: *m*_ is given by

$$\begin{array}{c} \begin{array}{cc} f_{k:m}\left(x\right) & =\frac{m!}{(k-1)!(m-k)!}{\left(F_{X}\left(x\right)\right)}^{k-1}{\left(1-F_{X}\left(x\right)\right)}^{m-k}f_{X}\left(x\right)\\ & =\frac{m!}{(k-1)!(m-k)!}2^{m-k+2}\lambda \alpha \sigma^{2}e^{-{\left(\frac{\sigma }{x}\right)}^{2}}{\left(1-e^{-{\left(\frac{\sigma }{x}\right)}^{2}}\right)}^{\alpha \lambda m-\alpha \lambda k+\alpha \lambda -1}\\ & \times \frac{{\left(1-{\left(1-e^{-{\left(\frac{\sigma }{x}\right)}^{2}}\right)}^{\alpha \lambda }\right)}^{k-1}}{x^{3}{\left(1+{\left(1-e^{-{\left(\frac{\sigma }{x}\right)}^{2}}\right)}^{\alpha \lambda }\right)}^{m+1}}\;. \end{array} \end{array}$$ (12)

Putting k = 1 in (12), we obtain pdf of the smallest order statistic as follows

$$\begin{array}{c} f_{1:m}(x)=\frac{2^{m+1}m\lambda \alpha \sigma^{2}e^{-{\left(\frac{\sigma }{x}\right)}^{2}}{\left(1+{\left(1-e^{-{\left(\frac{\sigma }{x}\right)}^{2}}\right)}^{\alpha \lambda }\right)}^{\alpha \lambda m-1}}{x^{3}{\left(1+{\left(1-e^{-{\left(\frac{\sigma }{x}\right)}^{2}}\right)}^{\alpha \lambda }\right)}^{m+1}}\;. \end{array}$$

The pdf of largest order statistic is obtained by putting k = m in (12) and is given by

$$\begin{array}{c} f_{m:m}\left(x\right)=\frac{4m\lambda \alpha \sigma^{2}e^{-{\left(\frac{\sigma }{x}\right)}^{2}}{\left(1-e^{-{\left(\frac{\sigma }{x}\right)}^{2}}\right)}^{\alpha \lambda -1}{\left(1-{\left(1-e^{-{\left(\frac{\sigma }{x}\right)}^{2}}\right)}^{\alpha \lambda }\right)}^{m-1}}{x^{3}{\left(1+{\left(1-e^{-{\left(\frac{\sigma }{x}\right)}^{2}}\right)}^{\alpha \lambda }\right)}^{m+1}}\;. \end{array}$$

### 3.7 Quantile and random number generation

Quantile are very needful for estimation purposes basically, quantile estimators and also it is used in simulation. The *p*^th^ quantile of the HLEIRD is given by

$$\begin{array}{c} Q\left(p\right)=\frac{\sigma }{\sqrt{-\text{ln}(1-{\left(\frac{1-p}{1+p}\right)}^{1/\lambda \alpha })}}. \end{array}$$ (13)

Quantile can also be used in finding the skewness and kurtosis of the distribution.

The equation (13) can be used in simulation to generate random variable from HLEIRD. Given U∼Uniform (0,1) then the random variable *X* from HLEIRD is given by

$$\begin{array}{c} X=\frac{\sigma }{\sqrt{-\text{ln}(1-{\left(\frac{1-\text{U}}{1+\text{U}}\right)}^{1/\lambda \alpha })}}. \end{array}$$

### 3.8 Median

Median of HLEIRD is obtained by substituting *p* = 1/2 in (13) and is given by

$$\begin{array}{c} \text{Median}=\frac{\sigma }{\sqrt{-\text{ln}(1-{\left(\frac{1}{3}\right)}^{1/\lambda \alpha })}}. \end{array}$$

### 3.9 Skewness and kurtosis using quantile approach

There are different methods which are used to find skewness and kurtosis in a certain distribution. The most common method is the one which use moments of the distribution but for HLEIRD we have first moment only. Due to this reason the appropriate approach of finding kurtosis and skewness is by using quantiles.

$$\text{skewness}=\frac{\left(Q_{3}-Q_{2})-(Q_{2}-Q_{1}\right)}{\left(Q_{2}-Q_{1})+(Q_{3}-Q_{2}\right)},$$

where *Q*_1_, *Q*_2_ and *Q*_3_ are obtained respectively by putting $p=\frac{1}{4}$, $p=\frac{1}{2}$ and $p=\frac{3}{4}$ in 13.

Kurtosis is known as the measure of dispersion of distribution. Moors (1988) suggested a robust alternative measure of kurtosis as follows:

$$\text{Kurtosis}=\frac{\left(E_{7}-E_{5})-(E_{3}-E_{1}\right)}{E_{6}-E_{2}},$$

where *E*_*i*_ is the *i*^th^ octile given by $E_{i}=Q(\frac{i}{8}).$

**Fig 5:**
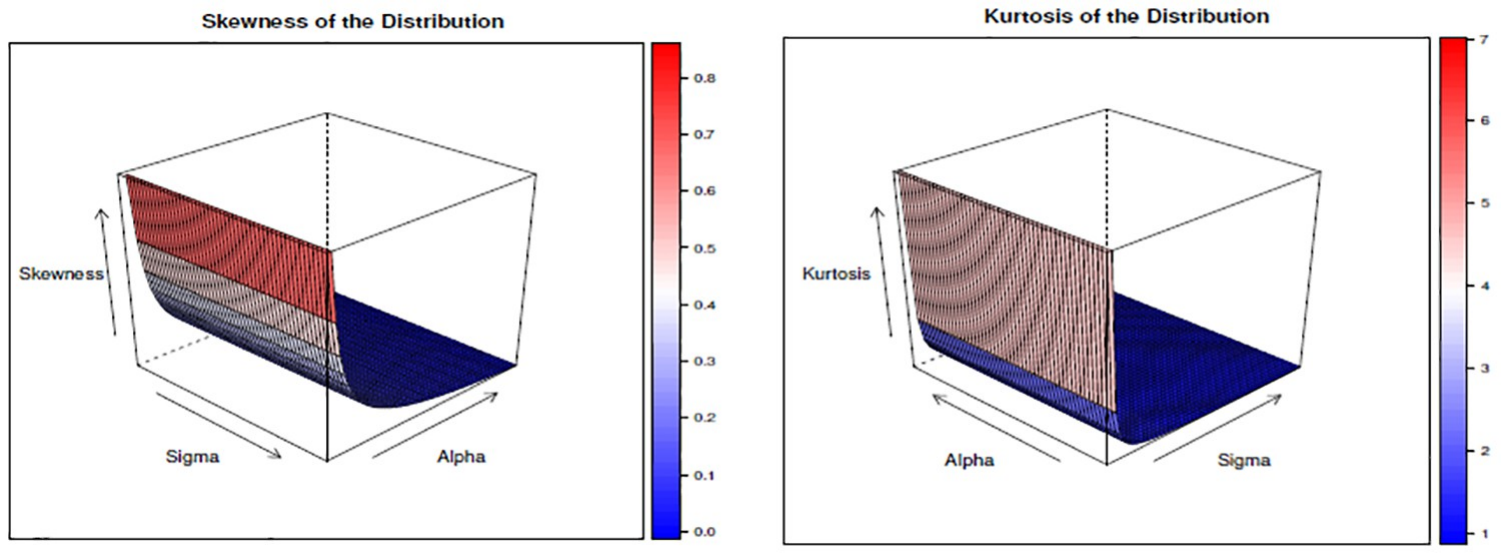
The skewness and kurtosis plot when λ = 2.

**Fig 6:**
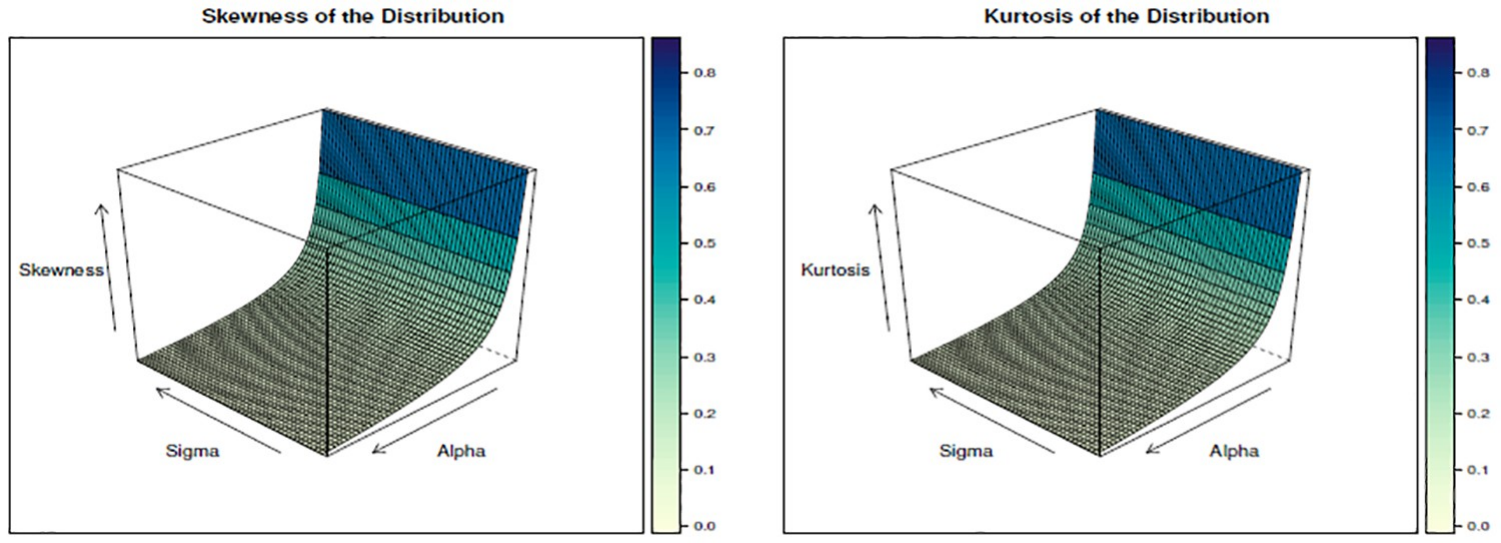
The skewness and kurtosis plot when *α* = 2.

**Fig 7:**
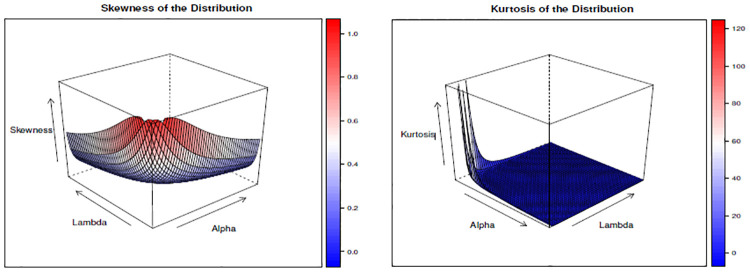
The skewness and kurtosis plot when *σ* = 2.

We have tabulated the value of skewness and kurtosis for different values of λ, *α*, and *σ* and given in Table 2. Similarly, we have drawn Figs 5–7 to show the changes in skewness and kurtosis for different values of λ, *α*, and *σ*. The findings show that when λ is constant increase in both *α*, and *σ* leads to a decrease in skewness and kurtosis. Likewise, for the case when *α* is constant, findings show that increases in both *σ* and λ leads to a decrease in skewness and kurtosis. Lastly, when *σ* is constant, the findings depict the same results as *α* and λ when they are constant, but in this case, it is found that the kurtosis is above 3 when *α*=0.5 and λ=0.5, which depicts the presence of leptokurtic. This result indicates that the kurtosis and skewness decrease when these parameters increase, and it reaches a point where the curve becomes a mesokurtic and platykurtic curve, as shown in Figs 5–7. Moreover, the finding depicts that the distribution is positively skewed (right-skewed) as it is evidenced by the presence of positive values for all skewness values calculated under different parametric values.

**Table 2:** The Galtons skewness and Moors kurtosis for some values of (*σ*, *α*,λ) of HLEIRD.

*σ*	*α*	λ	kurtosis	skewness	*σ*	*α*	λ	kurtosis	skewness
0.5	0.5	2.0	0.300056	1.54687	0.5	0.5	0.5	0.728443	4.52466
1.0	1.0	2.0	0.182664	1.35115	1.0	1.0	1.0	0.300056	1.54687
1.5	1.5	2.0	0.134317	1.30134	1.1	1.1	1.1	0.262377	1.47131
2.0	2.0	2.0	0.106396	1.27979	1.2	1.2	1.2	0.231820	1.41925
2.5	2.5	2.0	0.087638	1.26814	1.3	1.3	1.3	0.206583	1.38198
3.0	3.0	2.0	0.073903	1.26101	1.4	1.4	1.4	0.185402	1.35446
3.5	3.5	2.0	0.063269	1.25630	1.5	1.5	1.5	0.167371	1.33362
4.0	4.0	2.0	0.054709	1.25301	1.6	1.6	1.6	0.151832	1.31751
4.5	4.5	2.0	0.047617	1.25063	1.7	1.7	1.7	0.138295	1.30483
5.0	5.0	2.0	0.041611	1.24885	1.8	1.8	1.8	0.126389	1.29470
0.5	2.0	0.5	0.300056	1.54687	1.9	1.9	1.9	0.115830	1.28650
1.0	2.0	1.0	0.182664	1.35115	2.0	2.0	2.0	0.106396	1.27979
1.5	2.0	1.5	0.134317	1.30134	2.1	2.1	2.1	0.097910	1.27424
2.0	2.0	2.0	0.106396	1.27979	2.2	2.2	2.2	0.090232	1.26961
2.5	2.0	2.5	0.087638	1.26814	2.3	2.3	2.3	0.083248	1.26573
3.0	2.0	3.0	0.073903	1.26101	2.4	2.4	2.4	0.076864	1.26245
3.5	2.0	3.5	0.063269	1.25630	2.5	2.5	2.5	0.071004	1.25966
4.0	2.0	4.0	0.054709	1.25301	2.6	2.6	2.6	0.065602	1.25727
4.5	2.0	4.5	0.047617	1.25063	2.7	2.7	2.7	0.060605	1.25523
5.0	2.0	5.0	0.041611	1.24885	2.8	2.8	2.8	0.055967	1.25347
2.0	0.5	0.5	0.728443	4.52466	2.9	2.9	2.9	0.051649	1.25195
2.0	1.0	1.0	0.300056	1.54687	3.0	3.0	3.0	0.047617	1.25063
2.0	1.5	1.5	0.167371	1.33362	3.1	3.1	3.1	0.043842	1.24949
2.0	2.0	2.0	0.106396	1.27979	3.2	3.2	3.2	0.040300	1.24849
2.0	2.5	2.5	0.071004	1.25966	3.3	3.3	3.3	0.036968	1.24763
2.0	3.0	3.0	0.047617	1.25063	3.4	3.4	3.4	0.033828	1.24687
2.0	3.5	3.5	0.030862	1.24621	3.5	3.5	3.5	0.030862	1.24621
2.0	4.0	4.0	0.018181	1.24400	3.6	3.6	3.6	0.028056	1.24564
2.0	4.5	4.5	0.008195	1.24294	3.7	3.7	3.7	0.025395	1.24514
2.0	5.0	5.0	0.000093	1.24253	3.8	3.8	3.8	0.022870	1.24471

## 4 Methods of estimation

In this section, different methods for the estimation of parameters *σ*, *α* and λ of the HLEIRD are discussed. The various methods of estimation are maximum likelihood, ordinary and weighted least square, and percentile based estimation.

### 4.1 Maximum likelihood estimation

Let *X*_1_, *X*_2_, …, *X*_*n*_ be random sample of size n drawn from HLEIRD, then the likelihood function can be obtained as follows

$$\begin{array}{c} L=4^{n}\alpha^{n}\lambda^{n}\sigma^{2n}x^{-3n}e^{-\frac{n\sigma^{2}}{x^{2}}}{\left(1-e^{-\frac{\sigma^{2}}{x^{2}}}\right)}^{n(\alpha \lambda -1)}{\left({\left(1-e^{-\frac{\sigma^{2}}{x^{2}}}\right)}^{\alpha \lambda }+1\right)}^{-2n}. \end{array}$$ (14)

The log likelihood is obtained as

$$\begin{array}{c} \begin{array}{c} Log\left(L\right)=n\;\text{log}\left(\alpha \right)+n\;\text{log}\left(\lambda \right)+2n\;\text{log}\left(\sigma \right)+n\left(\alpha \lambda -1\right)\;\text{log}(1-e^{-\frac{\sigma^{2}}{x^{2}}})\\ -2n\;\text{log}({\left(1-e^{-\frac{\sigma^{2}}{x^{2}}}\right)}^{\alpha \lambda }+1)-\frac{n\sigma^{2}}{x^{2}}-3n\;\text{log}\left(x\right)+2n\;\text{log}\left(2\right). \end{array} \end{array}$$ (15)

Then

$$\frac{\partial log(L)}{\partial \lambda }=0$$

gives

$$\begin{array}{c} \frac{n}{\lambda }-\frac{2\alpha n\;\text{log}(1-e^{-\frac{\sigma^{2}}{x^{2}}}){\left(1-e^{-\frac{\sigma^{2}}{x^{2}}}\right)}^{\alpha \lambda }}{{\left(1-e^{-\frac{\sigma^{2}}{x^{2}}}\right)}^{\alpha \lambda }+1}+\alpha n\;\text{log}(1-e^{-\frac{\sigma^{2}}{x^{2}}})=0. \end{array}$$ (16)

Also

$$\frac{\partial log(L)}{\partial \alpha }=0$$

gives

$$\begin{array}{c} \frac{n}{\alpha }-\frac{2\lambda n\;\text{log}(1-e^{-\frac{\sigma^{2}}{x^{2}}}){\left(1-e^{-\frac{\sigma^{2}}{x^{2}}}\right)}^{\alpha \lambda }}{{\left(1-e^{-\frac{\sigma^{2}}{x^{2}}}\right)}^{\alpha \lambda }+1}+\lambda n\;\text{log}(1-e^{-\frac{\sigma^{2}}{x^{2}}})=0. \end{array}$$ (17)

and

$$\frac{\partial log(L)}{\partial \sigma }=0$$

gives

$$\begin{array}{c} \frac{2n}{\sigma }-\frac{4\alpha \lambda n\sigma e^{-\frac{\sigma^{2}}{x^{2}}}{\left(1-e^{-\frac{\sigma^{2}}{x^{2}}}\right)}^{\alpha \lambda -1}}{x^{2}({\left(1-e^{-\frac{\sigma^{2}}{x^{2}}}\right)}^{\alpha \lambda }+1)}+\frac{2n\sigma \left(\alpha \lambda -1\right)e^{-\frac{\sigma^{2}}{x^{2}}}}{x^{2}(1-e^{-\frac{\sigma^{2}}{x^{2}}})}-\frac{2n\sigma }{x^{2}}=0. \end{array}$$ (18)

By solving (16), (17) and (18) all together, we get the estimates of *σ*, *α* and λ. We can get the solution of the above equations by using methods like Newton Raphson method or bisection method. Also, ML estimators follows asymptotically normally distribution, that is $\sqrt{n}\left(\hat{\alpha }-\alpha ,\hat{\sigma }-\sigma ,\hat{\lambda }-\lambda \right)∼N_{3}\left(0,\Sigma \right)$, *Σ* is the dispersion matrix and is given by *Σ* = *I*^−1^, where

$$I=-E\left[\begin{array}{lll} \frac{\partial^{2}log\left(L\right)}{\partial \sigma^{2}} & \frac{\partial^{2}log\left(L\right)}{\partial \sigma \partial \alpha } & \frac{\partial^{2}log\left(L\right)}{\partial \sigma \partial \lambda }\\ \frac{\partial^{2}log\left(L\right)}{\partial \alpha \partial \sigma } & \frac{\partial^{2}log\left(L\right)}{\partial \alpha^{2}} & \frac{\partial^{2}log\left(L\right)}{\partial \alpha \partial \lambda }\\ \frac{\partial^{2}log\left(L\right)}{\partial \lambda \partial \sigma } & \frac{\partial^{2}log\left(L\right)}{\partial \lambda \partial \alpha } & \frac{\partial^{2}log\left(L\right)}{\partial \lambda^{2}} \end{array}\right],$$

$$\begin{array}{cc} \frac{\partial^{2}log\left(L\right)}{\partial \lambda^{2}} & =-\frac{n}{\lambda^{2}}-\frac{2\alpha^{2}\;n{\text{log}}^{2}(1-e^{-\frac{\sigma^{2}}{x^{2}}}){\left(1-e^{-\frac{\sigma^{2}}{x^{2}}}\right)}^{\alpha \lambda }}{{\left({\left(1-e^{-\frac{\sigma^{2}}{x^{2}}}\right)}^{\alpha \lambda }+1\right)}^{2}}, \end{array}$$ (19)

$$\begin{array}{cc} \frac{\partial^{2}log\left(L\right)}{\partial \alpha^{2}} & =-\frac{n}{\alpha^{2}}-\frac{2\lambda^{2}n\;{\text{log}}^{2}(1-e^{-\frac{\sigma^{2}}{x^{2}}}){\left(1-e^{-\frac{\sigma^{2}}{x^{2}}}\right)}^{\alpha \lambda }}{{\left({\left(1-e^{-\frac{\sigma^{2}}{x^{2}}}\right)}^{\alpha \lambda }+1\right)}^{2}},\; \end{array}$$ (20)

$$\begin{array}{c} \begin{array}{cc} \frac{\partial^{2}log\left(L\right)}{\partial \sigma^{2}} & =-\frac{2n}{\sigma^{2}}-\frac{4\alpha \lambda ne^{-\frac{\sigma^{2}}{x^{2}}}{\left(1-e^{-\frac{\sigma^{2}}{x^{2}}}\right)}^{\alpha \lambda -1}}{x^{2}({\left(1-e^{-\frac{\sigma^{2}}{x^{2}}}\right)}^{\alpha \lambda }+1)}+\frac{2n\left(\alpha \lambda -1\right)e^{-\frac{\sigma^{2}}{x^{2}}}}{x^{2}(1-e^{-\frac{\sigma^{2}}{x^{2}}})}\\ \; & +\frac{8\alpha^{2}\lambda^{2}n\sigma^{2}e^{-\frac{2\sigma^{2}}{x^{2}}}{\left(1-e^{-\frac{\sigma^{2}}{x^{2}}}\right)}^{2\alpha \lambda -2}}{x^{4}{\left({\left(1-e^{-\frac{\sigma^{2}}{x^{2}}}\right)}^{\alpha \lambda }+1\right)}^{2}}-\frac{4n\sigma^{2}\left(\alpha \lambda -1\right)e^{-\frac{2\sigma^{2}}{x^{2}}}}{x^{4}{\left(1-e^{-\frac{\sigma^{2}}{x^{2}}}\right)}^{2}}\\ \; & -\frac{2n}{x^{2}}+\frac{8\alpha \lambda n\sigma^{2}e^{-\frac{\sigma^{2}}{x^{2}}}{\left(1-e^{-\frac{\sigma^{2}}{x^{2}}}\right)}^{\alpha \lambda -1}}{x^{4}({\left(1-e^{-\frac{\sigma^{2}}{x^{2}}}\right)}^{\alpha \lambda }+1)}-\frac{4n\sigma^{2}\left(\alpha \lambda -1\right)e^{-\frac{\sigma^{2}}{x^{2}}}}{x^{4}(1-e^{-\frac{\sigma^{2}}{x^{2}}})}\\ \; & -\frac{8\alpha \lambda n\sigma^{2}\left(\alpha \lambda -1\right)e^{-\frac{2\sigma^{2}}{x^{2}}}{\left(1-e^{-\frac{\sigma^{2}}{x^{2}}}\right)}^{\alpha \lambda -2}}{x^{4}({\left(1-e^{-\frac{\sigma^{2}}{x^{2}}}\right)}^{\alpha \lambda }+1)}, \end{array} \end{array}$$ (21)

$$\begin{array}{c} \frac{\;\partial^{2}log\left(L\right)}{\partial \sigma \partial \alpha }=-\frac{2\lambda n\sigma ({\left(1-e^{-\frac{\sigma^{2}}{x^{2}}}\right)}^{2\alpha \lambda }+2\alpha \lambda \text{log}(1-e^{-\frac{\sigma^{2}}{x^{2}}}){\left(1-e^{-\frac{\sigma^{2}}{x^{2}}}\right)}^{\alpha \lambda }-1)}{x^{2}(e^{\frac{\sigma^{2}}{x^{2}}}-1){\left({\left(1-e^{-\frac{\sigma^{2}}{x^{2}}}\right)}^{\alpha \lambda }+1\right)}^{2}}, \end{array}$$ (22)

$$\begin{array}{c} \frac{\;\partial^{2}log\left(L\right)}{\partial \sigma \partial \lambda }=-\frac{2\alpha n\sigma ({\left(1-e^{-\frac{\sigma^{2}}{x^{2}}}\right)}^{2\alpha \lambda }+2\alpha \lambda \text{log}(1-e^{-\frac{\sigma^{2}}{x^{2}}}){\left(1-e^{-\frac{\sigma^{2}}{x^{2}}}\right)}^{\alpha \lambda }-1)}{x^{2}(e^{\frac{\sigma^{2}}{x^{2}}}-1){\left({\left(1-e^{-\frac{\sigma^{2}}{x^{2}}}\right)}^{\alpha \lambda }+1\right)}^{2}}, \end{array}$$ (23)

$$\begin{array}{c} \begin{array}{cc} \frac{\;\partial^{2}log\left(L\right)}{\partial \lambda \partial \alpha } & =-\frac{n\;\text{log}(1-e^{-\frac{\sigma^{2}}{x^{2}}})(2\alpha \lambda \text{log}(1-e^{-\frac{\sigma^{2}}{x^{2}}}){\left(1-e^{-\frac{\sigma^{2}}{x^{2}}}\right)}^{\alpha \lambda }-1)}{{\left({\left(1-e^{-\frac{\sigma^{2}}{x^{2}}}\right)}^{\alpha \lambda }+1\right)}^{2}}\\ \; & -\frac{n\;\text{log}(1-e^{-\frac{\sigma^{2}}{x^{2}}}){\left(1-e^{-\frac{\sigma^{2}}{x^{2}}}\right)}^{2\alpha \lambda }}{{\left({\left(1-e^{-\frac{\sigma^{2}}{x^{2}}}\right)}^{\alpha \lambda }+1\right)}^{2}}. \end{array} \end{array}$$ (24)

Then the asymptotic (1-*ξ*)100% confidence interval for any parameters *β* is given by

$$\left(\hat{\beta }-Z_{\xi /2}\sqrt{\Sigma_{ii}}\;,\;\hat{\beta }+Z_{\xi /2}\sqrt{\Sigma_{ii}}\right)$$

where *Σ*_*ii*_ is the diagonal element of matrix *Σ* and *Z*_*q*_ is the *q*^*th*^ quantile of a standard normal distribution.

### 4.2 Ordinary and weighted least-squares estimators

These least square methods were suggested by Swain et al. [16], for estimating parameters of Beta distribution. This study employed these estimation methods namely ordinary and weighted least-squares estimation method. Suppose *X*_1:*n*_, *X*_2:*n*_, ⋅⋅⋅, *X*_*n*: *n*_are the order statistics of a random sample of size n from HLEIRD with pdf given in (6). The least square estimators (LSEs) ${\hat{\sigma }}_{LSE}$, ${\hat{\alpha }}_{LSE}$ and ${\hat{\lambda }}_{LSE}$ of the parameters *σ*, *α* and λ are obtained by minimizing E with respect to *σ*, *α* and λ respectively.

where

$$\begin{array}{c} \text{E}=\sum_{i=1}^{n}{\left[F\left(x_{i:n}\right)-\frac{i}{n+1}\right]}^{2}. \end{array}$$ (25)

$$\begin{array}{c} =\sum_{i=1}^{n}{\left[\frac{1-{\left(1-e^{-{\left(\frac{\sigma }{x_{i:n}}\right)}^{2}}\right)}^{\alpha \lambda }}{1+{\left(1-e^{-{\left(\frac{\sigma }{x_{i:n}}\right)}^{2}}\right)}^{\alpha \lambda }}-\frac{i}{n+1}\right]}^{2}. \end{array}$$ (26)

Let

$$\begin{array}{c} \begin{array}{cc} \vartheta_{1}\left(x_{i:n}\right) & =\frac{\partial F(x_{i:n})}{\partial \sigma }\\ \; & =\frac{-\alpha \lambda {\left(1-e^{-{\left(\frac{\sigma }{x_{i:n}}\right)}^{2}}\right)}^{\alpha \lambda -1}2(\frac{\sigma }{x_{i:n}})e^{-{\left(\frac{\sigma }{x_{i:n}}\right)}^{2}}}{{\left(1+{\left(1-e^{-{\left(\frac{\sigma }{x_{i:n}}\right)}^{2}}\right)}^{\alpha \lambda }\right)}^{2}}\\ \; & \times (-2(\frac{\sigma }{x_{i:n}})e^{-{\left(\frac{\sigma }{x_{i:n}}\right)}^{2}}). \end{array} \end{array}$$ (27)

$$\begin{array}{c} \begin{array}{cc} \vartheta_{2}\left(x_{i:n}\right) & =\frac{\partial F(x_{i:n})}{\partial \alpha }\\ \; & =\frac{-\alpha \lambda {\left(1-e^{-{\left(\frac{\sigma }{x_{i:n}}\right)}^{2}}\right)}^{\alpha \lambda -1}2(\frac{\sigma }{x_{i:n}})e^{-{\left(\frac{\sigma }{x_{i:n}}\right)}^{2}}}{{\left(1+{\left(1-e^{-{\left(\frac{\sigma }{x_{i:n}}\right)}^{2}}\right)}^{\alpha \lambda }\right)}^{2}}\\ \; & \times \lambda {\left(\frac{\sigma }{x_{i:n}}\right)}^{2}{\left(1-e^{-{\left(\frac{\sigma }{x_{i:n}}\right)}^{2}}\right)}^{\alpha \lambda -1}. \end{array} \end{array}$$ (28)

and

$$\begin{array}{c} \begin{array}{cc} \vartheta_{3}\left(x_{i:n}\right) & =\frac{\partial F(x_{i:n})}{\partial \lambda }\\ \; & =\frac{-\alpha \lambda {\left(1-e^{-{\left(\frac{\sigma }{x_{i:n}}\right)}^{2}}\right)}^{\alpha \lambda -1}2(\frac{\sigma }{x_{i:n}})e^{-{\left(\frac{\sigma }{x_{i:n}}\right)}^{2}}}{{\left(1+{\left(1-e^{-{\left(\frac{\sigma }{x_{i:n}}\right)}^{2}}\right)}^{\alpha \lambda }\right)}^{2}}\\ \; & \times \alpha \;\text{log}(1-e^{-{\left(\frac{\sigma }{x_{i:n}}\right)}^{2}}){\left(1-e^{-{\left(\frac{\sigma }{x_{i:n}}\right)}^{2}}\right)}^{\alpha \lambda }. \end{array} \end{array}$$ (29)

By solving the following equations we get the LSEs of *σ*, *α* and λ respectively.

$$\begin{array}{c} \sum_{i=1}^{n}[\frac{1-{\left(1-e^{-{\left(\frac{\sigma }{x_{i:n}}\right)}^{2}}\right)}^{\alpha \lambda }}{1+{\left(1-e^{-{\left(\frac{\sigma }{x_{i:n}}\right)}^{2}}\right)}^{\alpha \lambda }}-\frac{i}{n+1}]\vartheta_{k}\left(x_{i:n}\right)=0\;,\;k=1,2,3. \end{array}$$ (30)

Next we consider the weighted least square estimators (WLSE).

Let

$$\begin{array}{c} Q=\sum_{i=1}^{n}[\frac{{\left(n+1\right)}^{2}\left(n+2\right)}{i(n-i+1)}{\left[F\left(x_{i:n}\right)-\frac{i}{n+1}\right]}^{2}] \end{array}$$

$$\begin{array}{c} =\sum_{i=1}^{n}[\frac{{\left(n+1\right)}^{2}\left(n+2\right)}{i(n-i+1)}{\left[\frac{1-{\left(1-e^{-{\left(\frac{\sigma }{x_{i:n}}\right)}^{2}}\right)}^{\alpha \lambda }}{1+{\left(1-e^{-{\left(\frac{\sigma }{x_{i:n}}\right)}^{2}}\right)}^{\alpha \lambda }}-\frac{i}{n+1}\right]}^{2}]. \end{array}$$ (31)

The weighted least square estimators can be obtained by minimizing *Q* in (31) with respect to unknown parameters *σ*, *α* and λ. Thus WLSE of *σ*, *α* and λ are respectively obtained by solving the following equations

$$\begin{array}{c} \sum_{i=1}^{n}[\frac{{\left(n+1\right)}^{2}\left(n+2\right)}{i(n-i+1)}[\frac{1-{\left(1-e^{-{\left(\frac{\sigma }{x_{i:n}}\right)}^{2}}\right)}^{\alpha \lambda }}{1+{\left(1-e^{-{\left(\frac{\sigma }{x_{i:n}}\right)}^{2}}\right)}^{\alpha \lambda }}-\frac{i}{n+1}]]\vartheta_{k}\left(x_{i:n}\right)=0,k=1,2,3. \end{array}$$ (32)

### 4.3 Cramér–Von Mises estimation

The selection of Cramér–Von Mises-type minimum distance estimators was supported by empirical evidence presented by MacDonald [17], which indicated that the estimator’s bias is smaller than that of the other minimum distance estimators.

Let

$$\begin{array}{c} CV=\frac{1}{12n}+\sum_{i=1}^{n}{\left[F\left(x_{i:n}\right)-\frac{2i-1}{2n}\right]}^{2} \end{array}$$

$$\begin{array}{c} =\frac{1}{12n}+\sum_{i=1}^{n}{\left[\frac{1-{\left(1-e^{-{\left(\frac{\sigma }{x_{i:n}}\right)}^{2}}\right)}^{\alpha \lambda }}{1+{\left(1-e^{-{\left(\frac{\sigma }{x_{i:n}}\right)}^{2}}\right)}^{\alpha \lambda }}-\frac{2i-1}{2n}\right]}^{2}. \end{array}$$ (33)

Cramér–Von Mises estimatiors of HLEIRD parameters are obtained by minimizing (33) with respect to *σ*, *α* and λ or by solving the following Eqs 34, 35 and 36 simultaneously.

$$\begin{array}{c} \sum_{i=1}^{n}[\frac{1-{\left(1-e^{-{\left(\frac{\sigma }{x_{i:n}}\right)}^{2}}\right)}^{\alpha \lambda }}{1+{\left(1-e^{-{\left(\frac{\sigma }{x_{i:n}}\right)}^{2}}\right)}^{\alpha \lambda }}-\frac{2i-1}{2n}]\vartheta_{1}\left(x_{i:n}\right)=0, \end{array}$$ (34)

$$\begin{array}{c} \sum_{i=1}^{n}[\frac{1-{\left(1-e^{-{\left(\frac{\sigma }{x_{i:n}}\right)}^{2}}\right)}^{\alpha \lambda }}{1+{\left(1-e^{-{\left(\frac{\sigma }{x_{i:n}}\right)}^{2}}\right)}^{\alpha \lambda }}-\frac{2i-1}{2n}]\vartheta_{2}\left(x_{i:n}\right)=0, \end{array}$$ (35)

$$\begin{array}{c} \sum_{i=1}^{n}[\frac{1-{\left(1-e^{-{\left(\frac{\sigma }{x_{i:n}}\right)}^{2}}\right)}^{\alpha \lambda }}{1+{\left(1-e^{-{\left(\frac{\sigma }{x_{i:n}}\right)}^{2}}\right)}^{\alpha \lambda }}-\frac{2i-1}{2n}]\vartheta_{3}\left(x_{i:n}\right)=0, \end{array}$$ (36)

where *ϑ*_1_(*x*_*i*: *n*_), *ϑ*_2_(*x*_*i*: *n*_) and *ϑ*_3_(*x*_*i*: *n*_) are defined in (27), (28) and (29) respectively.

### 4.4 Method of maximum product of spacings

The maximum product spacing (MPS) method was first introduced by Cheng and Amin [18, 19] as a substitute for the maximum likelihood method for the estimation of univariate continuous probability distributions. In addition to that, the MPS derived independently as an approximation to the Kullback-Leibler measure of information by Ranneby [20]. The method was proven efficient and consistent as MLE under more general conditions.

By refering the notation applied in section 4.2 the uniform spacings of a random sample from the HLEIRD distribution is defined as

$$D_{i}\left(\sigma ,\alpha ,\lambda \right)=F\left(x_{i:n}|\sigma ,\alpha ,\lambda \right)-F\left(x_{i-1:n}|\sigma ,\alpha ,\lambda \right),\;\;\;1,2,\cdots ,n,$$

where *F*(*x*_0_|*σ*, *α*, λ) = 0, *F*(*x*_*n* + 1: *n*_|*σ*, *α*, λ) = 1 and $\sum_{i=1}^{n+1}D_{i}\left(\sigma ,\alpha ,\lambda \right)=1$.

The maximum product spacings estimates ${\hat{\sigma }}_{MPS}$, ${\hat{\alpha }}_{MPS}$ and ${\hat{\lambda }}_{MPS}$ of the parameters *σ*, *α* and λ are obtained by maximizing *G*(*σ*, *α*, λ) with respect to *σ*, *α* and λ respectively, where

$$\begin{array}{c} G\left(\sigma ,\alpha ,\lambda \right)={\left[\prod_{i=1}^{n+1}D_{i}\left(\sigma ,\alpha ,\lambda \right)\right]}^{1/(n+1)}, \end{array}$$ (37)

$$\begin{array}{c} \text{Let}\;\;\;\;\;H\left(\sigma ,\alpha ,\lambda \right)=\frac{1}{n+1}\sum_{i=1}^{n+1}log[D_{i}\left(\sigma ,\alpha ,\lambda \right)] \end{array}$$ (38)

Then the estimates are obtained maximizing *H*(*σ*, *α*, λ) with respect to *σ*, *α* and λ respectively are obtained by or by solving the following Eqs 39, 40 and 41 simultaneously.

$$\begin{array}{c} \frac{\partial log(H(\sigma ,\alpha ,\lambda ))}{\partial \sigma }=\frac{1}{n+1}\sum_{i=1}^{n+1}\frac{1}{D_{i}\left(\sigma ,\alpha ,\lambda \right)}[\vartheta_{1}\left(x_{i:n}\right)-\vartheta_{1}\left(x_{i-1:n}\right)]=0, \end{array}$$ (39)

$$\begin{array}{c} \frac{\partial log(H(\sigma ,\alpha ,\lambda ))}{\partial \alpha }=\frac{1}{n+1}\sum_{i=1}^{n+1}\frac{1}{D_{i}\left(\sigma ,\alpha ,\lambda \right)}[\vartheta_{2}\left(x_{i:n}\right)-\vartheta_{2}\left(x_{i-1:n}\right)]=0, \end{array}$$ (40)

$$\begin{array}{c} \frac{\partial log(H(\sigma ,\alpha ,\lambda ))}{\partial \lambda }=\frac{1}{n+1}\sum_{i=1}^{n+1}\frac{1}{D_{i}\left(\sigma ,\alpha ,\lambda \right)}[\vartheta_{3}\left(x_{i:n}\right)-\vartheta_{3}\left(x_{i-1:n}\right)]=0, \end{array}$$ (41)

where *ϑ*_1_(.), *ϑ*_2_(.) and *ϑ*_3_(.) were defined in (27), (28) and (29) respectively.

## 5 Simulation study

In this section, a Monte Carlo simulation study is conducted to evaluate the performance of diferent estimation methods described in the previous sections. The performance of the diferent estimators is evaluated in terms of mean square error (MSE). The simulation is conducted by using R-sofware, 10000 random samples of size n from HLEIRD was generated for n = (20, 40, 70, 100) and (*σ*, *α*,λ) = (0.5,0.5,1), (1,0.5,1), (0.5,1,1), (1,1,1), (0.5,0.5,2), (1,0.5,2), (0,5,1,2), and (1,1,2).

Average bias and MSE values of the estimates obtained by the method of MLE, LSE, WLSE, CVME and MPS are shown in Tables 2–7. All methods show that they have consistency property since the values of average bias and MSE decrease as the sample size increases. Based on the value of MSE, the method of MLE shows good performance since it has small value of MSE compared to other methods.

**Table 3:** Average bias and MSE for maximum likelihood estimation.

(*σ*, *α*, λ)	n	BIAS			MSE		
(0.5, 0.5, 1)		*σ*	*α*	λ	*σ*	*α*	λ
	20	0.0524	0.0392	0.0302	0.0212	0.0210	0.0085
	40	0.0253	0.0115	0.0278	0.0084	0.0086	0.0043
	70	0.0133	0.0000	0.0283	0.0041	0.0044	0.0032
	100	0.0094	-0.0043	0.0279	0.0028	0.0030	0.0028
	150	0.0068	-0.0076	0.0290	0.0019	0.0020	0.0026
(1, 0.5, 1)	20	0.1056	0.0489	0.0205	0.0848	0.0251	0.0137
	40	0.0483	0.0171	0.0250	0.0323	0.0116	0.0085
	70	0.0288	0.0034	0.0279	0.0171	0.0063	0.0064
	100	0.0198	-0.0021	0.0291	0.0113	0.0044	0.0053
	150	0.0113	-0.0068	0.0312	0.0073	0.0032	0.0046
(0.5, 1, 1)	20	0.0335	-0.0045	0.1295	0.0106	0.0413	0.0490
	40	0.0157	-0.0259	0.0885	0.0045	0.0217	0.0208
	70	0.0098	-0.0311	0.0676	0.0024	0.0117	0.0115
	100	0.0064	-0.0351	0.0602	0.0016	0.0088	0.0089
	150	0.0045	-0.0334	0.0511	0.0011	0.0061	0.0063
(1, 1, 1)	20	0.0636	-0.0177	0.1433	0.0432	0.0402	0.0538
	40	0.0329	-0.0360	0.0978	0.0180	0.0215	0.0246
	70	0.0185	-0.0352	0.0741	0.0096	0.0130	0.0145
	100	0.0123	-0.0357	0.0614	0.0065	0.0099	0.0103
	150	0.0085	-0.0315	0.0493	0.0043	0.0071	0.0071
(0.5, 0.5, 2)	20	0.0333	0.0493	0.0634	0.0108	0.0331	0.0284
	40	0.0154	0.0128	0.0680	0.0045	0.0105	0.0136
	70	0.0094	0.0009	0.0694	0.0024	0.0052	0.0109
	100	0.0056	-0.0072	0.0731	0.0016	0.0034	0.0102
	150	0.0042	-0.0099	0.0730	0.0011	0.0023	0.0090
(1, 0.5, 2)	20	0.0645	0.0540	0.0541	0.0417	0.0353	0.0293
	40	0.0324	0.0150	0.0633	0.0181	0.0118	0.0181
	70	0.0171	0.0002	0.0699	0.0094	0.0055	0.0135
	100	0.0116	-0.0054	0.0702	0.0064	0.0036	0.0119
	150	0.0093	-0.0076	0.0676	0.0043	0.0023	0.0098
(0.5, 1, 2)	20	0.0251	0.1297	0.0923	0.0067	0.1868	0.0533
	40	0.0122	0.0412	0.0741	0.0029	0.0627	0.0236
	70	0.0065	0.0059	0.0704	0.0016	0.0282	0.0168
	100	0.0046	-0.0030	0.0673	0.0011	0.0184	0.0150
	150	0.0032	-0.0121	0.0654	0.0007	0.0127	0.0139
(1, 1, 2)	20	0.0501	0.1352	0.0928	0.0272	0.2021	0.0655
	40	0.0250	0.0477	0.0679	0.0118	0.0706	0.0273
	70	0.0134	0.0151	0.0621	0.0063	0.0318	0.0204
	100	0.0090	0.0022	0.0599	0.0044	0.0217	0.0174
	150	0.0070	-0.0065	0.0596	0.0029	0.0135	0.0155

**Table 4:** Average bias and MSE for ordinary least square estimation.

(*σ*, *α*, λ)	n	BIAS			MSE		
(0.5, 0.5, 1)		*σ*	*α*	λ	*σ*	*α*	λ
	20	0.0015	0.0018	0.0385	0.0257	0.0305	0.0222
	40	0.0002	-0.0016	0.0275	0.0115	0.0145	0.0140
	70	0.0001	-0.0041	0.0224	0.0063	0.0076	0.0103
	100	0.0009	-0.0053	0.0193	0.0044	0.0052	0.0076
	150	0.0006	-0.0077	0.0213	0.0029	0.0030	0.0061
(1, 0.5, 1)	20	0.0009	0.0093	0.0475	0.1052	0.0421	0.0399
	40	-0.0021	0.0005	0.0316	0.0466	0.0197	0.0231
	70	0.0011	-0.0038	0.0289	0.0246	0.0101	0.0128
	100	0.0002	-0.0072	0.0296	0.0172	0.0070	0.0098
	150	-0.0001	-0.0101	0.0292	0.0117	0.0048	0.0077
(0.5, 1, 1)	20	-0.0052	-0.0484	0.0825	0.0134	0.0758	0.0557
	40	-0.0029	-0.0490	0.0654	0.0060	0.0380	0.0275
	70	-0.0017	-0.0425	0.0554	0.0034	0.0232	0.0180
	100	-0.0014	-0.0374	0.0480	0.0023	0.0164	0.0146
	150	-0.0009	-0.0333	0.0395	0.0015	0.0113	0.0100
(1, 1, 1)	20	-0.0129	-0.0554	0.0959	0.0517	0.0877	0.0600
	40	-0.0075	-0.0488	0.0703	0.0236	0.0452	0.0323
	70	-0.0018	-0.0381	0.0568	0.0136	0.0290	0.0218
	100	-0.0012	-0.0336	0.0482	0.0095	0.0206	0.0168
	150	-0.0018	-0.0318	0.0407	0.0061	0.0150	0.0128
(0.5, 0.5, 2)	20	-0.0048	0.0040	0.0656	0.0126	0.0378	0.0523
	40	-0.0035	-0.0050	0.0546	0.0060	0.0154	0.0356
	70	-0.0017	-0.0078	0.0525	0.0033	0.0082	0.0250
	100	-0.0007	-0.0093	0.0545	0.0024	0.0051	0.0193
	150	-0.0004	-0.0099	0.0517	0.0016	0.0034	0.0147
(1, 0.5, 2)	20	-0.0099	0.0080	0.0745	0.0517	0.0483	0.0747
	40	-0.0046	-0.0041	0.0623	0.0240	0.0180	0.0486
	70	-0.0057	-0.0097	0.0586	0.0138	0.0085	0.0350
	100	-0.0027	-0.0101	0.0598	0.0093	0.0055	0.0275
	150	-0.0009	-0.0107	0.0544	0.0060	0.0034	0.0199
(0.5, 1, 2)	20	-0.0043	0.0263	0.0797	0.0083	0.2064	0.1379
	40	-0.0034	0.0012	0.0578	0.0039	0.0925	0.0772
	70	-0.0016	-0.0044	0.0461	0.0022	0.0438	0.0550
	100	-0.0020	-0.0097	0.0382	0.0015	0.0288	0.0399
	150	-0.0013	-0.0130	0.0424	0.0010	0.0184	0.0353
(1, 1, 2)	20	-0.0141	0.0214	0.0808	0.0335	0.2274	0.1392
	40	-0.0068	0.0086	0.0586	0.0156	0.0993	0.0819
	70	-0.0037	-0.0042	0.0472	0.0087	0.0476	0.0582
	100	-0.0032	-0.0070	0.0417	0.0060	0.0323	0.0477
	150	-0.0019	-0.0123	0.0460	0.0040	0.0189	0.0389

**Table 5:** Average bias and MSE for weighted least square estimation.

(*σ*, *α*, λ)	n	BIAS			MSE		
(0.5, 0.5, 1)		*σ*	*α*	λ	*σ*	*α*	λ
	20	0.0007	-0.0013	0.0436	0.0324	0.0297	0.0250
	40	0.0033	0.0010	0.0282	0.0142	0.0150	0.0141
	70	0.0019	-0.0022	0.0222	0.0077	0.0075	0.0104
	100	0.0029	-0.0036	0.0211	0.0054	0.0051	0.0076
	150	0.0016	-0.0049	0.0181	0.0035	0.0032	0.0058
(1, 0.5, 1)	20	0.0115	0.0130	0.0464	0.1333	0.0454	0.0449
	40	0.0068	0.0045	0.0324	0.0589	0.0227	0.0250
	70	0.0029	-0.0029	0.0301	0.0311	0.0125	0.0167
	100	0.0052	-0.0050	0.0310	0.0214	0.0082	0.0124
	150	0.0028	-0.0086	0.0326	0.0140	0.0051	0.0095
(0.5, 1, 1)	20	-0.0043	-0.0468	0.0860	0.0151	0.0774	0.0525
	40	-0.0008	-0.0409	0.0685	0.0069	0.0364	0.0278
	70	0.0002	-0.0381	0.0555	0.0037	0.0224	0.0178
	100	0.0005	-0.0346	0.0470	0.0026	0.0153	0.0124
	150	0.0001	-0.0318	0.0411	0.0017	0.0106	0.0093
(1, 1, 1)	20	-0.0099	-0.0516	0.0908	0.0571	0.0756	0.0676
	40	-0.0029	-0.0399	0.0707	0.0277	0.0445	0.0344
	70	0.0001	-0.0377	0.0587	0.0154	0.0249	0.0223
	100	-0.0013	-0.0316	0.0475	0.0103	0.0196	0.0172
	150	-0.0001	-0.0287	0.0398	0.0070	0.0129	0.0108
(0.5, 0.5, 2)	20	-0.0053	0.0050	0.0591	0.0148	0.0398	0.0438
	40	-0.0008	-0.0009	0.0553	0.0068	0.0149	0.0273
	70	0.0002	-0.0061	0.0554	0.0039	0.0075	0.0193
	100	0.0004	-0.0082	0.0516	0.0025	0.0043	0.0146
	150	0.0001	-0.0078	0.0460	0.0017	0.0029	0.0121
(1, 0.5, 2)	20	-0.0075	0.0138	0.0563	0.0596	0.0546	0.0739
	40	-0.0031	0.0040	0.0418	0.0269	0.0182	0.0477
	70	-0.0029	-0.0029	0.0417	0.0152	0.0089	0.0296
	100	0.0001	-0.0058	0.0460	0.0104	0.0055	0.0238
	150	0.0006	-0.0068	0.0459	0.0069	0.0034	0.0177
(0.5, 1, 2)	20	-0.0070	0.0270	0.0751	0.0087	0.2289	0.1065
	40	-0.0024	0.0098	0.0505	0.0041	0.0878	0.0598
	70	-0.002	-0.0009	0.0395	0.0023	0.0418	0.0382
	100	-0.0013	-0.0080	0.0382	0.0015	0.0251	0.0308
	150	-0.0005	-0.0057	0.0295	0.0010	0.0154	0.0213
(1, 1, 2)	20	-0.0142	0.0172	0.0827	0.0338	0.1997	0.1209
	40	-0.0041	0.0143	0.0484	0.0171	0.1005	0.0757
	70	-0.0013	0.0080	0.0302	0.0087	0.0497	0.0489
	100	-0.0004	-0.0003	0.0358	0.0062	0.0304	0.0436
	150	-0.0002	-0.0033	0.0304	0.0042	0.0179	0.0305

**Table 6:** Average bias and MSE for Cramér–Von mises estimation.

(*σ*, *α*, λ)	n	BIAS			MSE		
(0.5, 0.5, 1)		*σ*	*α*	λ	*σ*	*α*	λ
	20	0.0547	0.0521	0.0319	0.0350	0.0426	0.0230
	40	0.0262	0.0231	0.0245	0.0133	0.0174	0.0130
	70	0.0149	0.0102	0.0209	0.0071	0.0105	0.0096
	100	0.0102	0.0028	0.0216	0.0046	0.0055	0.0075
	150	0.0070	-0.0019	0.0220	0.0030	0.0033	0.0057
(1, 0.5, 1)	20	0.1097	0.0648	0.0324	0.1436	0.0569	0.0462
	40	0.0533	0.0294	0.0215	0.0538	0.0246	0.0251
	70	0.0288	0.0119	0.0232	0.0272	0.0128	0.0134
	100	0.0207	0.0051	0.0242	0.0185	0.0087	0.0108
	150	0.0156	-0.0011	0.0251	0.0123	0.0054	0.0074
(0.5, 1, 1)	20	0.0326	0.0272	0.1227	0.0162	0.1021	0.0771
	40	0.0162	-0.0104	0.0853	0.0066	0.0398	0.0360
	70	0.0078	-0.0227	0.0651	0.0035	0.0227	0.0200
	100	0.0064	-0.0247	0.0557	0.0025	0.0162	0.0147
	150	0.0037	-0.0258	0.0455	0.0016	0.0107	0.0107
(1, 1, 1)	20	0.0663	0.0193	0.1399	0.0622	0.0950	0.0902
	40	0.0303	-0.0087	0.0859	0.0260	0.0504	0.0370
	70	0.0170	-0.0147	0.0645	0.0141	0.0291	0.0233
	100	0.0140	-0.0168	0.0529	0.0098	0.0218	0.0172
	150	0.0092	-0.0201	0.0445	0.0064	0.0136	0.0122
(0.5, 0.5, 2)	20	0.0336	0.0689	0.0715	0.0159	0.0597	0.0635
	40	0.0173	0.0244	0.0610	0.0069	0.0184	0.0358
	70	0.0098	0.0088	0.0563	0.0036	0.0090	0.0222
	100	0.0060	0.0005	0.0556	0.0024	0.0056	0.0181
	150	0.0040	-0.0040	0.0537	0.0016	0.0034	0.0145
(1, 0.5, 2)	20	0.0670	0.0733	0.0634	0.0643	0.0706	0.0794
	40	0.0322	0.0248	0.0580	0.0268	0.0224	0.0435
	70	0.0168	0.0056	0.0595	0.0139	0.0093	0.0304
	100	0.0118	0.0010	0.0580	0.0098	0.0065	0.0249
	150	0.0083	-0.0037	0.0572	0.0063	0.0037	0.0194
(0.5, 1, 2)	20	0.0232	0.1548	0.1198	0.0098	0.3387	0.1880
	40	0.0112	0.0659	0.0715	0.0043	0.1115	0.0811
	70	0.0069	0.0310	0.0530	0.0023	0.0532	0.0487
	100	0.0043	0.0121	0.0502	0.0015	0.0300	0.0401
	150	0.0028	0.0017	0.0485	0.0010	0.0204	0.0322
(1, 1, 2)	20	0.0497	0.1821	0.1101	0.0395	0.3997	0.2414
	40	0.0241	0.0763	0.0640	0.0166	0.1259	0.0842
	70	0.0122	0.0311	0.0508	0.0092	0.0523	0.0533
	100	0.0094	0.0177	0.0510	0.0063	0.0408	0.0479
	150	0.0049	0.0011	0.0495	0.0040	0.0209	0.0359

**Table 7:** Average bias and MSE for maximum product spacing method.

(*σ*, *α*, λ)	n	BIAS			MSE		
(0.5, 0.5, 1)		*σ*	*α*	λ	*σ*	*α*	λ
	20	-0.0394	-0.0496	0.0505	0.0156	0.0170	0.0119
	40	-0.0286	-0.0388	0.0388	0.0074	0.0084	0.0066
	70	-0.0210	-0.0319	0.0331	0.0041	0.0049	0.0046
	100	-0.0172	-0.0287	0.0326	0.0029	0.0035	0.0040
	150	-0.0137	-0.0251	0.0320	0.0019	0.0026	0.0037
(1, 0.5, 1)	20	-0.0751	-0.0482	0.0627	0.0638	0.0205	0.0207
	40	-0.0577	-0.0432	0.0553	0.0304	0.0110	0.0128
	70	-0.0442	-0.0377	0.0510	0.0170	0.0070	0.0095
	100	-0.0346	-0.0332	0.0482	0.0114	0.0052	0.0082
	150	-0.0270	-0.0289	0.0442	0.0074	0.0037	0.0065
(0.5, 1, 1)	20	-0.0338	-0.1444	0.0843	0.0093	0.0633	0.0247
	40	-0.0248	-0.1101	0.0613	0.0045	0.0347	0.0135
	70	-0.0175	-0.0858	0.0492	0.0025	0.0201	0.0086
	100	-0.0136	-0.0744	0.0439	0.0017	0.0143	0.0072
	150	-0.0102	-0.0597	0.0381	0.0011	0.0094	0.0055
(1, 1, 1)	20	-0.0687	-0.1526	0.0984	0.0361	0.0652	0.0291
	40	-0.0496	-0.1206	0.0778	0.0184	0.0389	0.0185
	70	-0.0343	-0.0934	0.0614	0.0098	0.0237	0.0121
	100	-0.0290	-0.0762	0.0498	0.0068	0.0167	0.0088
	150	-0.0211	-0.0623	0.0400	0.0045	0.0112	0.0067
(0.5, 0.5, 2)	20	-0.0356	-0.0520	0.0735	0.0091	0.0196	0.0246
	40	-0.0244	-0.0441	0.0751	0.0045	0.0098	0.0177
	70	-0.0177	-0.0378	0.0736	0.0025	0.0058	0.0139
	100	-0.0143	-0.0350	0.0755	0.0017	0.0042	0.0118
	150	-0.0107	-0.0311	0.0770	0.0011	0.0030	0.0106
1, 0.5, 2	20	-0.0683	-0.0499	0.0879	0.0372	0.0220	0.0358
	40	-0.0496	-0.0459	0.0864	0.0179	0.0108	0.0249
	70	-0.0348	-0.0371	0.0781	0.0098	0.0061	0.0182
	100	-0.0281	-0.0332	0.0755	0.0069	0.0044	0.0150
	150	-0.0205	-0.0284	0.0703	0.0046	0.0030	0.0124
(0.5, 1, 2)	20	-0.0303	-0.1115	0.0809	0.0062	0.1054	0.0487
	40	-0.0213	-0.0935	0.0676	0.0030	0.0501	0.0260
	70	-0.0153	-0.0791	0.0650	0.0017	0.0287	0.0214
	100	-0.0119	-0.0685	0.0636	0.0011	0.0211	0.0195
	150	-0.0088	-0.0577	0.0614	0.0008	0.0153	0.0183
(1, 1, 2)	20	-0.0605	-0.1114	0.0893	0.0241	0.1092	0.0552
	40	-0.0407	-0.0882	0.0700	0.0123	0.0534	0.0335
	70	-0.0298	-0.0745	0.0638	0.0070	0.0313	0.0272
	100	-0.0225	-0.0642	0.0619	0.0047	0.0225	0.0245
	150	-0.0186	-0.0576	0.0625	0.0031	0.0162	0.0225

The findings in Table 8 show the different ranks of the estimation method of HLEIRD from the different parametric combinations. The result showed that the MLE method outperforms all other estimation methods with an overall score of 54, which is less compared to all other estimation methods. Therefore, as evidenced by the simulation study, the MLE method performs best for HLEIRD.

**Table 8:** Ranks of all the methods of estimation for different parametric specifications based MSE.

(*σ*, *α*, λ)	n	MLE	LSE	WLS	CV	MPS
(0.5, 0.5, 1)	20	2	3	4	5	1
	40	2	3	5	4	1
	70	1	3	5	4	2
	100	1	4	4	4	2
	150	1	3	5	4	2
(1, 0.5, 1)	20	2	3	4	5	1
	40	2	3	4	5	1
	70	1	3	5	4	2
	100	1	3	5	4	2
	150	1	3	5	4	2
(0.5, 1, 1)	20	2	3	4	5	1
	40	2	3	4	5	1
	70	1	4	3	5	2
	100	1	4	3	5	2
	150	2	4	3	5	1
(1, 1, 1)	20	2	3	4	5	1
	40	1	3	4	5	2
	70	1	3	4	5	2
	100	1	3	4.5	4.5	2
	150	1	5	3	4	2
(0.5, 0.5, 2)	20	2	3	4	5	1
	40	2	4	3	5	1
	70	1	4	3	5	2
	100	1	4	3	5	2
	150	1	5	3	4	2
(1, 0.5, 2)	20	2	3	4	5	1
	40	2	3	5	4	1
	70	1	3	4	5	2
	100	1	3	4	5	2
	150	1	4	3	5	2
(0.5, 1, 2)	20	2	3	4	5	1
	40	1	4	3	5	2
	70	1	4	3	5	2
	100	1	4	3	5	2
	150	1	4	3	5	2
(1, 1, 2)	20	2	4	3	5	1
	40	1	3	4	5	2
	70	1	3.5	3.5	4	2
	100	1	4	3	5	2
	150	1	4	3	5	2
∑Rank		54	139.5	151	188.5	66
Overall rank		1	3	4	5	2

## 6 Application

In this section, we fit the proposed HLEIRD to real data sets and compare them with their sub-models such as exponentiated inverse Rayleigh distribution(EIRD), inverse Rayleigh distribution(IRD),Rayleigh distribution(RD) and inverse weibull distribution(IWD) distributions, respectively. The first data set represents the strength measured in GPA for single-carbon fibers and impregnated 1000-carbon fiber tows. The single fibers were tested under tension at a gauge of 20 mm. Data were initially reported by Badar and Priest (1982) and later applied by Kundu et al. [21].

The data is: 1.312,1.314, 1.479, 1.552, 1.700, 1.803, 1.861, 1.865, 1.944, 1.958, 1.966, 1.997, 2.006, 2.021, 2.027, 2.055, 2.063, 2.098, 2.14, 2.179, 2.224, 2.240, 2.253, 2.270, 2.272, 2.274, 2.301, 2.301, 2.359, 2.382, 2.382, 2.426, 2.434, 2.435, 2.478, 2.490, 2.511, 2.514, 2.535, 2.554, 2.566, 2.57, 2.586, 2.629, 2.633, 2.642, 2.648, 2.684, 2.697, 2.726, 2.770, 2.773, 2.800, 2.809, 2.818, 2.821, 2.848, 2.88, 2.954, 3.012, 3.067, 3.084, 3.090, 3.096, 3.128, 3.233, 3.433, 3.585, 3.585.

The second data set was extracted from Dey et al. [22], and the data set represents the strength measured in GPA for single-carbon fibers and impregnated 1000-carbon fiber tows, but for this case, single fibers were tested under tension at a gauge of 10 mm.

The data is: 0.562, 0.564, 0.729, 0.802, 0.950, 1.053, 1.111, 1.115,1.194, 1.208, 1.216, 1.247, 1.256, 1.271, 1.277, 1.305, 1.313, 1.348, 1.390, 1.429, 1.474, 1.490, 1.503, 1.520, 1.522, 1.524, 1.551, 1.551, 1.609, 1.632, 1.632, 1.676, 1.684, 1.685, 1.728, 1.740, 1.761, 1.764, 1.785, 1.804, 1.816, 1.824, 1.836, 1.879, 1.883, 1.892, 1.898, 1.934, 1.947, 1.976, 2.020, 2.023, 2.050, 2.059, 2.068, 2.071, 2.098, 2.130, 2.204, 2.262, 2.317, 2.334, 2.340, 2.346, 2.378, 2.483, 2.683, 2.835, 2.835.

### 6.1 Model validity and selection criteria

The Akaike information criteria (AIC), Consistent Akaike information iriterion (CAIC), Hannan-Quinn information criterion (HQIC), and Bayesian information criteria (BIC) are employed to verify that this HLEIRD model is appropriate for the datasets that have been taken. In addition to that, K-S distance and P-value were employed to verify the suitability of the model over the other four probability distribution models employed.

The expression of AIC, CAIC, HQIC and BIC are given by

$$\begin{array}{c} \text{AIC}=-2L\hat{\left(\theta \right)}+2q \end{array}$$ (42)

$$\begin{array}{c} \text{CAIC}=-2L\hat{\left(\theta \right)}+q\left(\text{log(n)}+1\right), \end{array}$$ (43)

$$\begin{array}{c} HQIC=-2L\hat{\left(\theta \right)}+2q\text{log}(\text{log(n)}), \end{array}$$ (44)

and

$$\begin{array}{c} BIC=-2L\hat{\left(\theta \right)}+q\text{log(n)}, \end{array}$$ (45)

where $L\hat{\left(\theta \right)}$ denotes the log-likelihood at MLEs, q is the number of parameters, and n is the sample size.

Tables 13 and 14 provide the goodness-of-fit measures, and their corresponding P-values for the fitted models of the two data sets employed in this study.

Table 9 show summary of the data set one which depict that the median is 2.478 which is higher than mean 2.451 this indicate presence of negative skewness. Additionally, this evidenced the skewness -0.0282. Also data is Platykurtic since the kurtosis is less than 3. Therefore, these findings provide a picture that the data is asymmetric in nature and it has a heavier tail to the left side.

**Table 9:** Summary statistics for data set one.

n	Min.	1st Qu.	Median	Mean	3rd Qu.	Max.	var	Skewness	kurtosis
69	1.312	2.098	2.478	2.451	2.773	3.585	0.2452	-0.0282	2.9407

**Fig 8:**
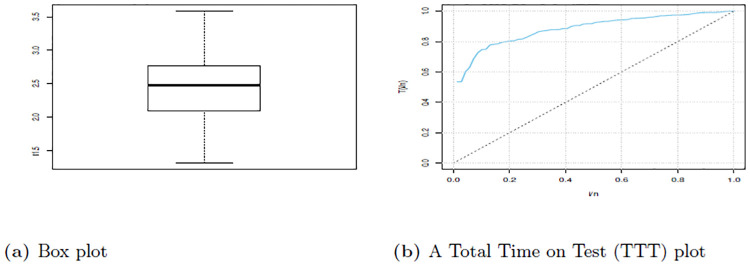
Box plot and TTT plot for data set one.

**Fig 9:**
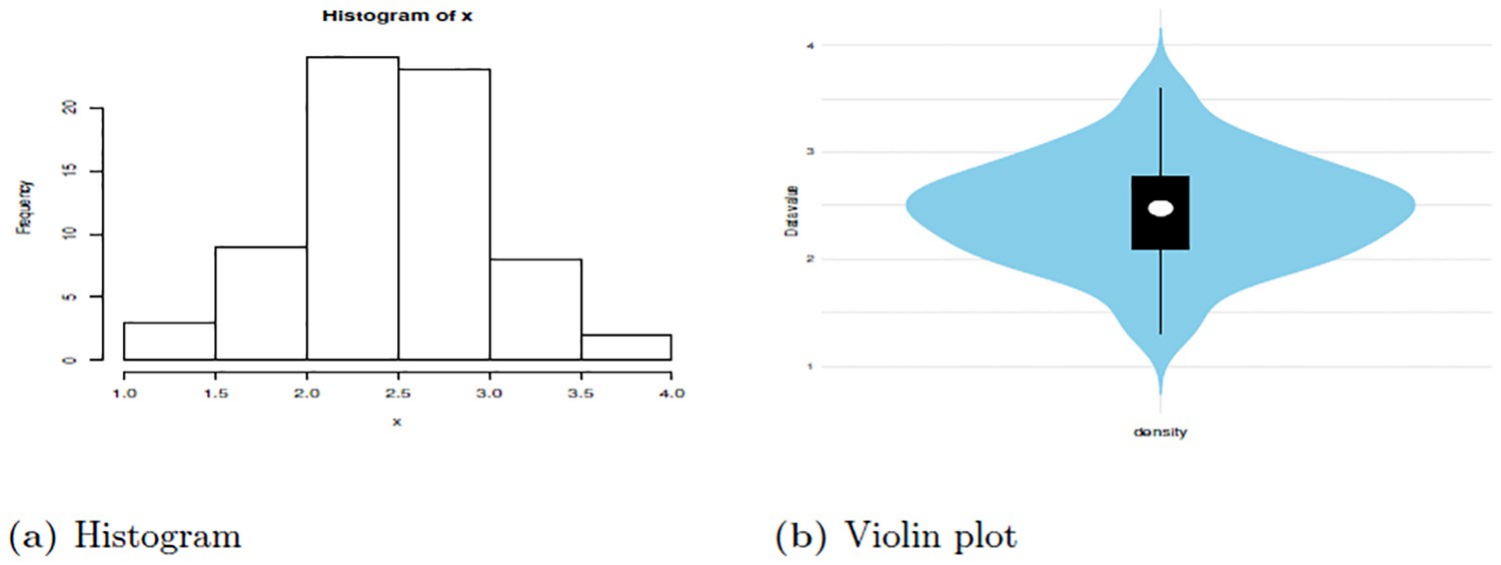
The histogram and violin plots for data set one.

Figs 8 and 9 give the Violin plot, Histogram, and Box plot for data set one, which show that there is a presence of left-skewness. This is evidenced by the Box plot and Violin plot. Also, if the Histogram plot covers more left part, indicates left-skewed presence. The TTT plot is concave, indicating a decreasing failure rate. Early failures are more frequent, and the rate of failures decreases over time. This is evidenced by a plot bends downward, indicating a decreasing failure rate. This suggests that the data set is appropriate for additional study since, as it was observed from the pdf plot, HLEIRD is capable of modeling asymmetric data or skewed data sets.

Table 10 shows summary of the data set one which depict that the median is 1.728 which is higher than mean 1.701 this the indicate presence of negative skewness. Additionally this evidenced the skewness -0.0285. Also data is Platykurtic since the kurtosis is less than 3. Therefore, these findings provide a picture that the data is asymmetric in nature and it has a heavier tail to the left side.

**Table 10:** Summary statistics for data set two.

n	Min.	1st Qu.	Median	Mean	3rd Qu.	Max.	var	Skewness	kurtosis
69	0.562	1.348	1.728	1.701	2.023	2.835	0.2452	-0.0285	2.9404

**Fig 10:**
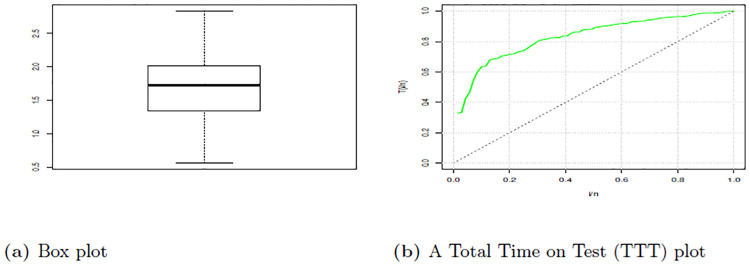
Box plot and TTT plot for data set two.

**Fig 11:**
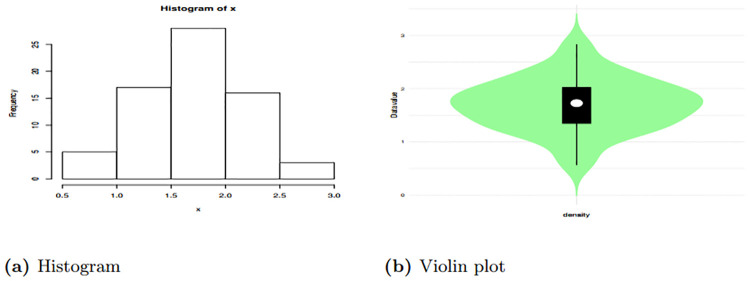
The histogram and violin plots for data set two.

Figs 10 and 11 give the Violin plot, Histogram, and Box plot for data set one, which show that there is the presence of a left-skewed. This is evidenced by the Box plot and Violin plot. Also, if the Histogram plot covers more left part, indicates left-skewed presence. The TTT plot is concave, indicating a decreasing failure rate. Early failures are more frequent, and the rate of failures decreases over time. This is evidenced by a plot bends downward, indicating a decreasing failure rate. This suggests that the data set is appropriate for additional study since, as it was observed from the pdf plot, HLEIRD is capable of modeling asymmetric data or skewed data sets.

The values in Tables 11 and 12 provide a comparison of the new distribution HLEIRD with the existing four distributions: EIRD, IRD, RD, and IWD. The findings in Table 11 show that HLEIRD has a low AIC,HQIC compared to all probability distributions employed, while in the case of BIC and CAIC, the EIRD has a lower BIC and CAIC. Since AIC,HQIC are more powerful criterion for choosing a model, for the first data set, HLEIRD was selected as the best model compared to the other models. In line with that, for the case of data set two, from Table 12, the HLEIRD was selected to be the best model compared to the other four distributions, EIRD, IRD, and IWD, since the HLEIRD was found to have a lower BIC, CAIC, HQIC, and AIC.

**Table 11:** Statistics of the MLEs, standard errors (SE) and criteria for model selection for data set one.

Model	Parameter	Estimates (SE)	-2l	Criteria				Rank
				AIC	CAIC	BIC	HQIC	
HLEIRD	*α*	0.7691(0.2828)	-50.5020	107.004	126.409	113.707	109.663	1
	*σ*	3.6338(0.2075)						
	λ	12.5518(4.6149)						
EIRD	*α*	3.9543(0.22261)	-52.0685	108.137	121.074	112.605	109.910	2
	*σ*	10.2893(2.85018)						
IRD	*α*	2.2828(0.13741)	-88.4131	178.826	185.294	181.060	179.713	3
RD	*α*	2.5001(0.15049)	-135.074	272.147	278.616	274.381	273.034	5
IWD	*α*	2.1437(0.06656)	-63.6236	131.247	144.184	135.715	133.020	4
	*σ*	4.1262(0.33816)						

**Table 12:** Statistics of the MLEs, standard errors (SE) and criteria for model selection for data set two.

Model	Parameter	Estimates (SE)	-2l	Criteria				Rank
				AIC	CAIC	BIC	HQIC	
HLEIRD	*α*	0.3379012(0.1428197)	-58.7619	123.524	142.752	130.1387	126.141	1
	*σ*	1.7974251(0.1253355)						
	λ	9.6244856(4.0680535)						
EIRD	*α*	2.52032 (0.534833)	-63.0921	130.184	143.003	134.594	131.929	2
	*σ*	1.84828(0.131619)						
IRD	*α*	1.40306(0.0857057)	-72.2784	146.557	152.966	148.761	147.429	4
RD	*α*	1.77604(0.108489)	-111.601	225.203	231.612	227.408	226.075	5
IWD	*α*	2.41960(0.06656)	-63.6236	143.707	156.526	148.116	145.452	3
	*σ*	1.34982(0.33816)						

### 6.2 Model goodness of fit

**Fig 12:**
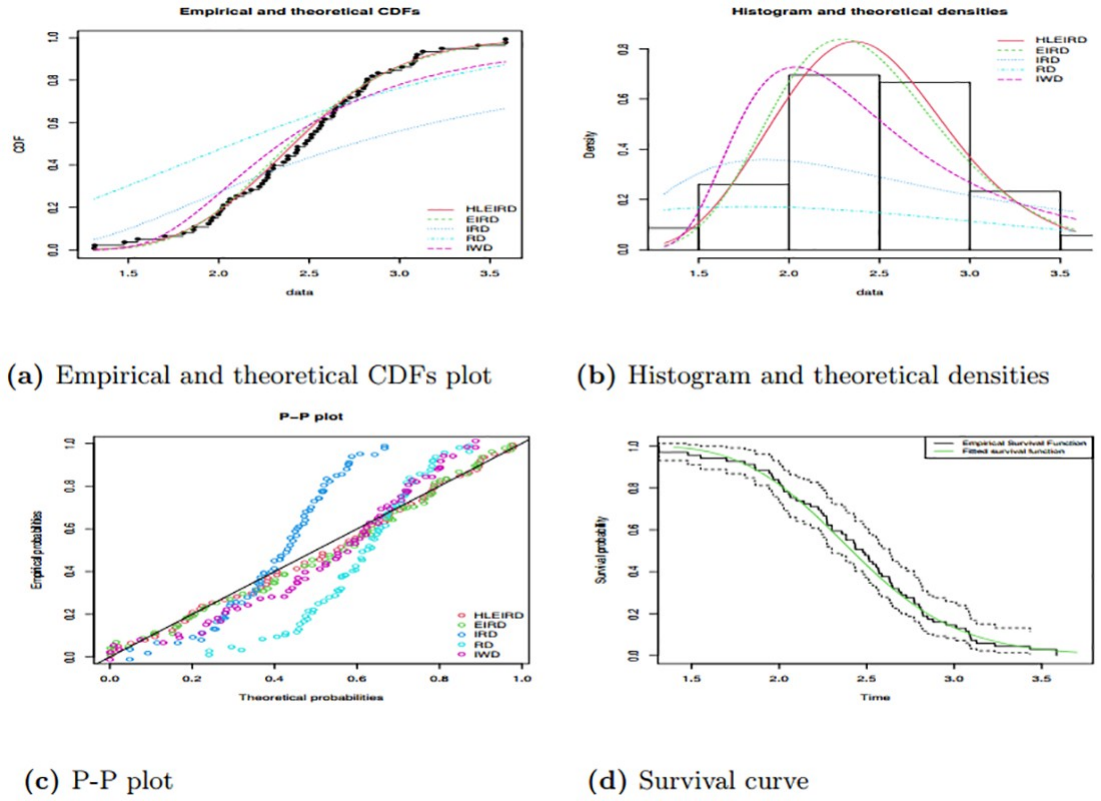
Shows empirical and theoretical CDFs, histogram and theoretical densities, P-P and survival plots for the data set one.

**Fig 13:**
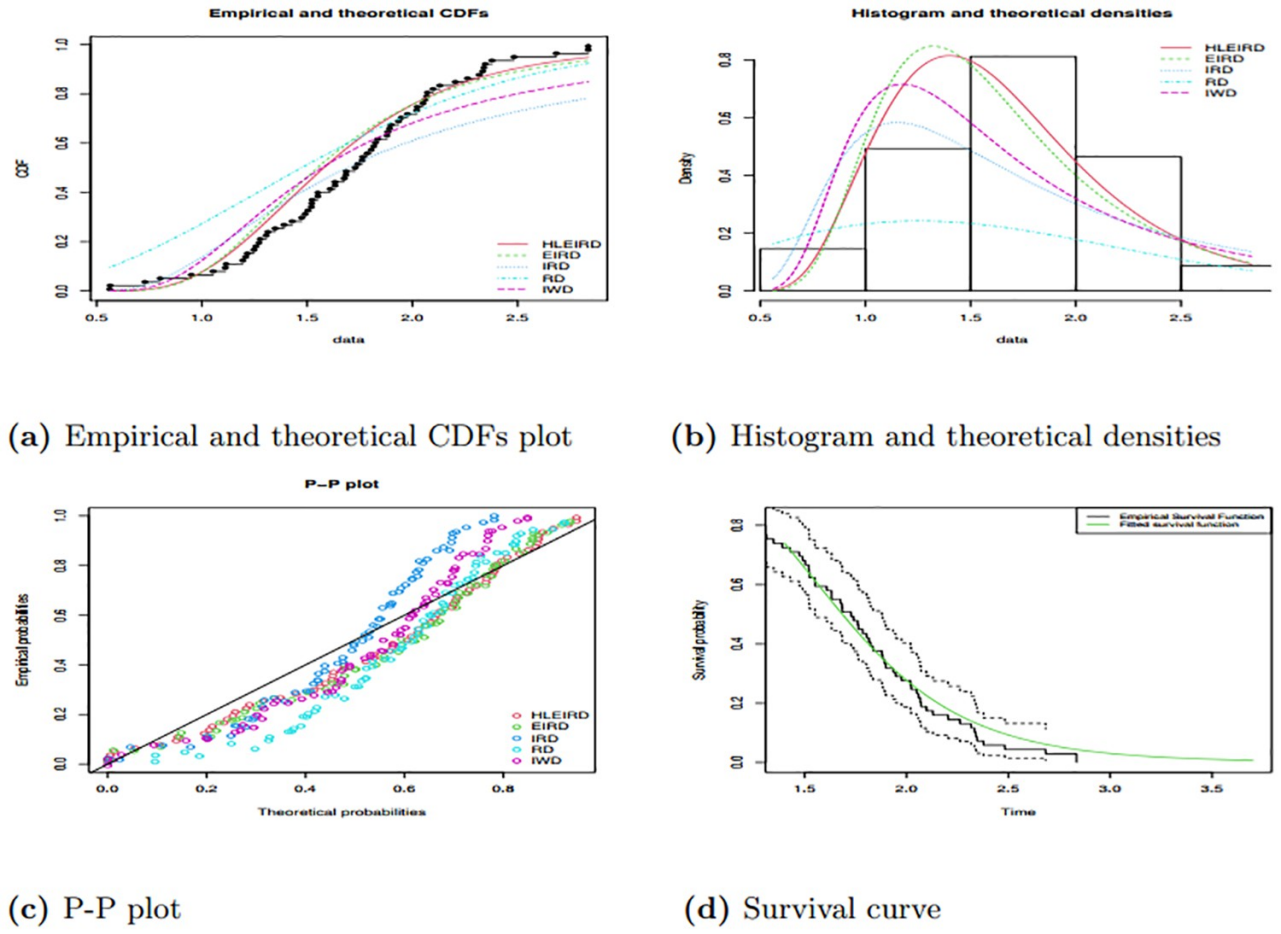
Shows empirical and theoretical CDFs, histogram and theoretical densities, P-P and survival plots for the data set two.

The results for Tables 13 and 14 show goodness of fit for five pdf employed in this study. Similarly, Figs 12 and 13 show the goodness of fit for all pdf employed in this study. The finding in Tables 13 and 14 show that the HLEIRD was fitting the data well compared to other pdf employed, since HLEIRD has the highest P-values for both data sets applied under all goodness fit of tests employed. Moreover, the graphs of PP plot, histogram, and theoretical densities and lastly, empirical and theoretical CDFs given in Figs 12 and 13 verify how well the used data set fits the HLEIRD compared with the other four distributions.

**Table 13:** Statistics of the goodness-of-fit for data set one.

Model	goodness of fit statistics						Rank
	*A* ^2^	P-value	*W*	P-value	K-S	P-value	
HLEIRD	0.3657	0.882	0.04372	0.915	0.05862	0.972	1
EIRD	0.6276	0.622	0.08599	0.659	0.07772	0.799	2
IRD	11.29	0.00000898	2.263	0.00000239	0.3549	5.63E-08	4
RD	12.01	0.00000871	2.393	0.00000116	0.3384	0.000000273	5
IWD	2.725	0.038	0.4139	0.0661	0.1337	0.17	3

**Table 14:** Statistics of the goodness-of-fit for data set two.

Model	goodness of fit statistics						Rank
	*A* ^2^	P-value	*W*	P-value	K-S	P-value	
HLEIRD	1.912	0.103	0.3093	0.127	0.1362	0.166	1
EIRD	100	0.00000896	10.25	0.0000	0.5677	0.0000	5
IRD	5.157	0.00244	0.868	0.00487	0.2343	0.00128	3
RD	6.075	0.000906	1.157	0.000992	0.2442	0.000676	4
IWD	3.941	0.00939	0.6283	0.0188	0.1647	0.0529	2

The Tables 15 and 16 give the estimated values of the parameters of HLEIRD using five different estimation methods: maximum likelihood, least square, weighted least square, maximum product spacings, and Cramer-von Mises estimation. The results show that under various estimation methods for data set one and data set two yields the same result as least square method of estimation fit well two both data sets employed. This is because of that the least squares estimation have the highest P-value in the case of the K-S test and the lowest likelihood value.

**Table 15:** The goodness of fit statistics for parameter estimated under various estimation methods for data set one.

Model	*α*	*σ*	λ	-2l	K-S	P-value
MLE	0.769110	3.633846	12.551840	50.5021	0.05862	0.9720
LSE	0.780379	3.719519	13.532278	0.0213694	0.04223	1.0000
WLSE	0.84883	3.79850	13.76247	297.41	0.04877	0.9970
CVME	0.836176	3.766375	13.411439	0.0214533	0.04542	0.9990
MPS	0.751411	3.527084	12.005041	4.67264	0.08371	0.7120

**Table 16:** The goodness of fit statistics for parameter estimated under various estimation methods for data set two.

Model	*α*	*σ*	λ	-2l	K-S	P-value
MLE	0.337901	1.797425	9.624486	58.7619	0.1362	0.166
LSE	0.365779	2.013078	10.972752	0.0628995	0.05636	0.974
WLSE	0.581977	2.181946	8.943666	799.135	0.08546	0.712
CVME	0.386406	2.043814	10.898757	0.0630516	0.0621	0.958
MPS	0.235156	1.639238	11.662787	4.80971	0.1422	0.126

## 7 Conclusion

In this paper, a new three-parameter probability distribution was introduced by applying a half-logistic transformation to the exponential inverse Rayleigh distribution. It is called the half-logistic exponential inverse Rayleigh distribution. Some of its statistical and mathematical properties such as stochastic ordering results, linear representation of HLEIRD, raw moments, moment generating function, entropy measure, order statistics, skewness and kurtosis features, quantile, and median, were derived. To estimate the parameters of the proposed distribution, five estimation methods were discussed. Specifically, simulations were performed to evaluate the performance of these estimation methods for both small and large samples through Monte Carlo simulations study. It revealed that the maximum likelihood was the best estimation compared to other estimation methods. In addition, the performance of the proposed distribution was assessed by fitting it to two actual data sets. The findings revealed that the new distribution performs better than alternative versions of the Rayleigh distributions. The limitation of this study is that the model may not be suitable for a very small sample size as well as high peaked data. Furthermore, future studies can develop bivariate or multivariate versions of HLEIRD to study engineering and medical problems in different dimensions. Lastly, this study employed only the classical estimation method, so the future study can employ the Bayesian estimation method to estimate the parameters of this newly proposed model.

## Supporting information

S1 Data set (DOCX)

S1 File (ZIP)
