# Supplementary material for: Half logistic exponentiated inverse Rayleigh distribution: Properties and application to life time data
Source: PLoS One. 2025 Jan 16;20(1):e0310681. doi: 10.1371/journal.pone.0310681 (PMC11737768; doi:10.1371/journal.pone.0310681)
Supplement: S1 Data set — (DOCX) [file pone.0310681.s001.docx]

**DATA SETS**

**FIRST DATA SET**

The first data set represents the strength measured in GPA for single-carbon fibers and impregnated 1000-carbon fiber tows. The single fibers were tested under tension at a gauge of 20 mm. Data were initially reported by Badar and Priest (1982) and later applied by Kundu and Raqab (2009).

The data is:

1.312,1.314, 1.479, 1.552, 1.700, 1.803, 1.861, 1.865, 1.944, 1.958, 1.966, 1.997, 2.006, 2.021, 2.027, 2.055,2.063, 2.098, 2.14, 2.179, 2.224, 2.240, 2.253, 2.270, 2.272, 2.274, 2.301, 2.301, 2.359, 2.382, 2.382, 2.426,2.434, 2.435, 2.478, 2.490, 2.511, 2.514, 2.535, 2.554, 2.566, 2.57, 2.586, 2.629, 2.633, 2.642, 2.648, 2.684,2.697, 2.726, 2.770, 2.773, 2.800, 2.809, 2.818, 2.821, 2.848, 2.88, 2.954, 3.012, 3.067, 3.084, 3.090, 3.096,3.128, 3.233, 3.433, 3.585, 3.585.

**SECOND DATA SET**

The second data set was extracted from Dey and Kundu (2014) and the data set represents the strength measured in GPA for single-carbon fibers and impregnated 1000-carbon fiber tows, but for this case, single fibers were tested under tension at a gauge of 10 mm.

The data is:

0.562, 0.564, 0.729, 0.802, 0.950, 1.053, 1.111, 1.115,1.194, 1.208,1.216, 1.247, 1.256, 1.271, 1.277, 1.305, 1.313, 1.348, 1.390, 1.429,1.474, 1.490,1.503, 1.520, 1.522, 1.524, 1.551,1.551, 1.609, 1.632,1.632, 1.676, 1.684, 1.685, 1.728, 1.740, 1.761, 1.764, 1.785, 1.804,1.816 ,1.824,1.836 ,1.879, 1.883, 1.892, 1.898, 1.934 ,1.947,1.976,2.020, 2.023, 2.050, 2.059, 2.068, 2.071, 2.098, 2.130, 2.204, 2.262,2.317, 2.334, 2.340, 2.346, 2.378, 2.483, 2.683, 2.835, 2.835.
